# Evolutionary dynamics of the chloroplast genome sequences of six *Colobanthus* species

**DOI:** 10.1038/s41598-020-68563-5

**Published:** 2020-07-13

**Authors:** Piotr Androsiuk, Jan Paweł Jastrzębski, Łukasz Paukszto, Karol Makowczenko, Adam Okorski, Agnieszka Pszczółkowska, Katarzyna Joanna Chwedorzewska, Ryszard Górecki, Irena Giełwanowska

**Affiliations:** 1 Department of Plant Physiology, Genetics and Biotechnology, Faculty of Biology and Biotechnology, University of Warmia and Mazury in Olsztyn, ul. M. Oczapowskiego 1A, 10-719 Olsztyn, Poland; 2 Department of Entomology, Phytopathology and Molecular Diagnostics, Faculty of Environmental Management and Agriculture, University of Warmia and Mazury in Olsztyn, ul. Prawocheńskiego 17, 10-720 Olsztyn, Poland; 3 Department of Agronomy, Warsaw University of Life Sciences-SGGW, ul. Nowoursynowska 166, 02-787 Warsaw, Poland

**Keywords:** Plant genetics, Comparative genomics, Next-generation sequencing

## Abstract

The complete plastome sequences of six species were sequenced to better understand the evolutionary relationships and mutation patterns in the chloroplast genome of the genus *Colobanthus*. The length of the chloroplast genome sequences of *C. acicularis*, *C. affinis*, *C. lycopodioides*, *C. nivicola*, *C. pulvinatus* and *C. subulatus* ranged from 151,050 to 151,462 bp. The quadripartite circular structure of these genome sequences has the same overall organization and gene content with 73 protein-coding genes, 30 tRNA genes, four rRNA genes and five conserved chloroplast open reading frames. A total of 153 repeat sequences were revealed. Forward repeats were dominant, whereas complementary repeats were found only in *C. pulvinatus*. The mononucleotide SSRs composed of A/T units were most common, and hexanucleotide SSRs were detected least often. Eleven highly variable regions which could be utilized as potential markers for phylogeny reconstruction, species identification or phylogeography were identified within *Colobanthus* chloroplast genomes. Seventy-three protein-coding genes were used in phylogenetic analyses. Reconstructed phylogeny was consistent with the systematic position of the studied species, and the representatives of the same genus were grouped in one clade. All studied *Colobanthus* species formed a single group and *C. lycopodioides* was least similar to the remaining species.

## Introduction

The genus *Colobanthus* in the family Caryophyllaceae contains 26 species^1^. Most species are found in the Southern Hemisphere, and the greatest diversity is observed in New Zealand^2^. *Colobanthus* species are low-growing perennials with a cushion growth habit, narrow and dense leaves, and inconspicuous, solitary, greenish flowers without petals, but with four to six prominent sepals^3,4^. The relationships within the family Caryophyllaceae are not easy to elucidate, partly due to arbitrarily and poorly defined genera and difficulties in determining phylogenetically useful morphological characters^5^. *Colobanthus* is one of the least studied genera, and it is sometimes confused with the related genus *Sagina* (Caryophyllaceae)^6^. Many areas where *Colobanthus* species occur are under protection, and some of them, such as the cold-temperate South Pacific Islands, have world heritage status^7^. Moreover, in contrast to the widespread species of *C. quitensi,* C. *affinis* and C. *apetalus*, taxa such as *C. strictus*, *C*. *squarrosus* and *C. curtisiae* (recorded in only three Tasmanian populations)^8–10^ or *C*. *nivicola* (endemic to the alpine tract of the Mt Kosciusko area in New South Wales, Australia)^11^ are extremely rare and are on the Australian list of rare or threatened plant species^12^. In many areas of Australia, where the habitats of *Colobanthus* species overlap, species such as *C. nivicola and C. pulvinatus* may be difficult to distinguish in the field^11^. Therefore, a precise identification of these species is necessary.

The members of the genus *Colobanthus* are extremely rarely studied, excluding Antarctic pearlwort (*Colobanthus quitensis* (Kunth) Bartl) which rose to fame as the only native representative of Magnoliopsida in the maritime Antarctic^13^. *Colobanthus quitensis* has been intensively studied to explore the traits responsible for its high tolerance to extreme Antarctic conditions^14–19^. Despite the above, our knowledge of the genetic diversity of this species and the entire genus *Colobanthus* remains limited^20–24^. Only two papers presented the complete sequence of the chloroplast genomes of *C. quitensis*^25^ and *C. apetalus*^26^, whereas the size of the nuclear genome in *C. quitensis* was estimated by flow cytometry in only one study^27^.

In recent years, chloroplast genome sequences attracted significant interest in plant phylogenetics, phylogeography and molecular evolution research^28^. Chloroplast genome sequences have numerous advantages, including low molecular weight, simple structure, uniparental (generally maternal) mode of inheritance, haploidy, highly conserved structure and a slower evolutionary rate of change than nuclear genomes. For this reason, chloroplast genomes constitute valuable data that are relatively easy to handle with source molecular data, support the validation of complex evolutionary relationships and detailed phylogenetic analyses at group, family or even genus level^29–32^. Chloroplast sequences also have numerous applications in biotechnology^33,34^ and the development of molecular markers for identifying species and distinguishing morphologically similar species^35–37^. A high number of new chloroplast genomes have been reported ever since the complete chloroplast genome sequences of *Nicotiana tabacum*^38^ and *Marchantia polymorpha*^39^ were published in 1986. This phenomenal progress was made possible by the development of new high-throughput genome sequencing technologies which enable scientists to obtain high quality cp genome sequences in a more convenient and relatively inexpensive way. As a result, around 3,500 chloroplast genomes have been deposited in the database of the National Center for Biotechnology Information (NCBI). It has been recently proposed that the whole chloroplast genome sequence should be used as a universal super-barcode in the identification of plant species. This approach may overcome the limitations of the traditional two-locus barcode based mainly on sequence variation within two plastome regions (*rbcL* and *matK*) that is not always sufficient for species discrimination^40^. In *Colobanthus*, the development of cpDNA markers has so far been restricted to the following loci: *matK*, *trnK* (*C. masonae*, *C. affinis*^41^), *matK*, *trnK*, *trnL*, *trnL–trnF*, *trnF*, *rps16* (*C. muscoides*; unpublished data, available in NCBI) and *ndhF* (*C. brevisepalus*^42^). Complete chloroplast genome sequences are available only for *C. quitensis*^25^ and *C. apetalus*^26^. The published data indicate that the *Colobanthus* cp genome is typical for angiosperms in terms of size (151,276 bp for *C. quitensis* and 151,228 bp for *C. apetalus*) and composition (112 genes). This genome has a conserved quadripartite circular structure with a large single copy (LSC) region, a small single copy (SSC) region and two copies of inverted repeat (IR) regions^43,44^. The complete sequences of the cp genome of *C. quitensis* and *C. apetalus* provide molecular data that are essential for advanced genomic studies. However, the genomic data for other members of the genus *Colobanthus* are still limited.

The complete chloroplast genomes of six *Colobanthus* species have been sequenced and annotated for the first time in this study. The comparative study of these six chloroplast genomes as well as the previously published cp genomes for *C. quitensis* and *C. apetalus* had the following goals: (1) to determine the size and structure of *Colobanthus* cp genomes, (2) to identify genomic repeats, including forward, reverse, palindromic and complementary sequences among *Colobanthus* genomes, (3) to compare the variation of simple sequence repeats (SSRs) among *Colobanthus* cp genomes, (4) to verify the phylogenetic relationships among *Colobanthus* species and other Caryophyllaceae species for which complete chloroplast genomes are available.

## Results

### Organization of chloroplast genomes

**Figure 1:**
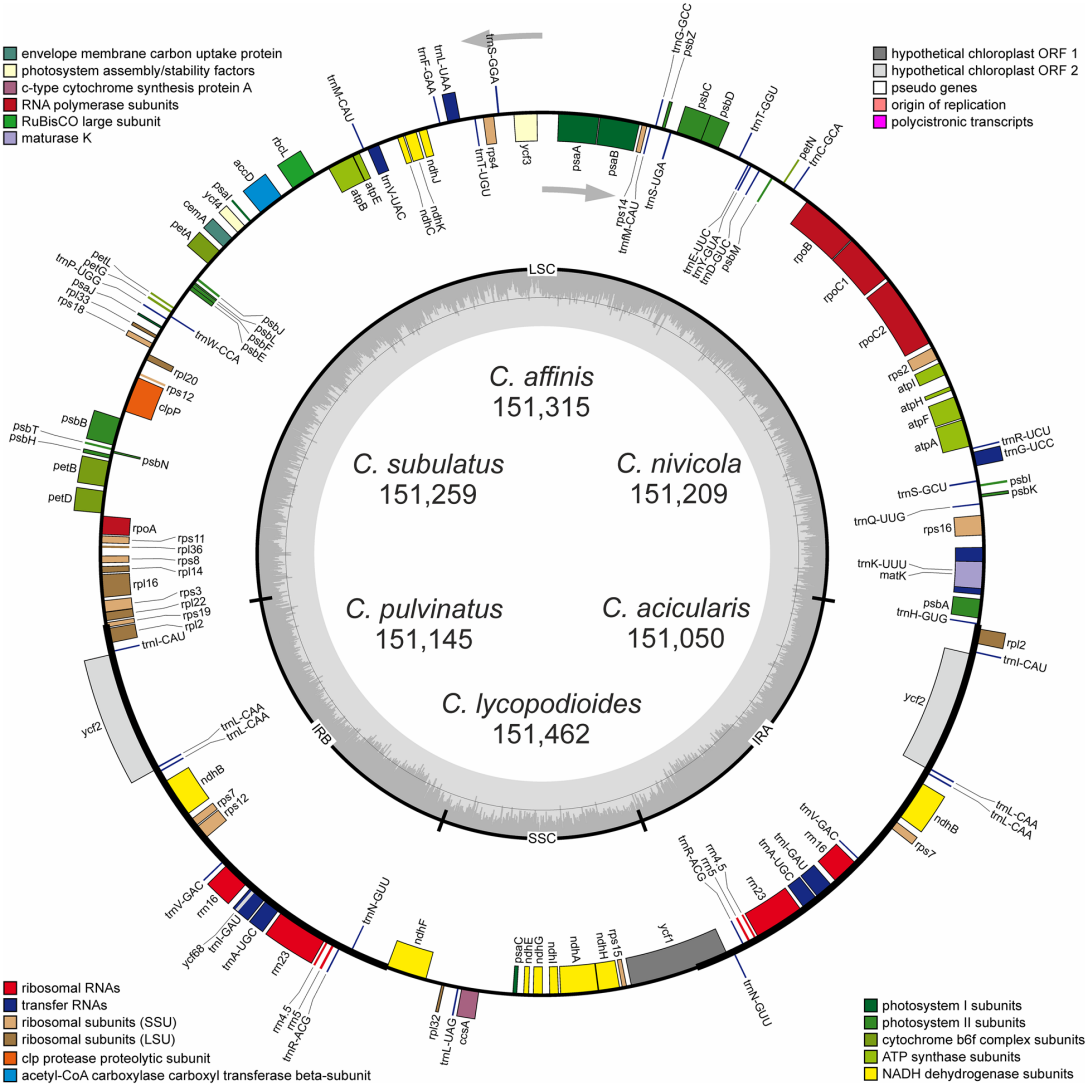
Gene map of the six *Colobanthus* chloroplast genomes. Genes drawn inside the circle are transcribed clockwise, and those outside are transcribed counterclockwise (indicated by arrows). Differential functional gene groups are color-coded. GC content variations is shown in the middle circle. Gene map was generated with the OrganellarGenomeDRAW (OGDRAW) 1.3.1. (https://chlorobox.mpimp-golm.mpg.de/OGDraw.html.)

Six *Colobanthus species* were sequenced to produce 1,986,760–7,849,218 raw reads (150 bp for average read length) which were mapped separately to the reference genome of *C. quitensis*. A total of 210,882–414,160 reads were ultimately mapped with 202.7× to 396.5 × coverage (Table 1). The six *Colobanthus* cp genome sequences were deposited in GenBank under the following accession numbers: MN273320 for *C. acicularis*, MN273318 for *C. affinis*, MN273317 for *C lycopodioides*, MN273316 for *C. nivicola*, MN273315 for *C. pulvinatus,* and MN273319 for *C. subulatus*. The chloroplast genome sequences described in this study ranged from 151,050 (*C. acicularis*) to 151,462 bp (*C. lycopodioides*). Each chloroplast genome was assembled into a single circular, double-stranded DNA sequence. All plastomes displayed a typical quadripartite structure with a pair of IRs (25,309–25,326 bp) separated by SSC (17,177–17,256 bp) and LSC (83,198–83,645 bp) regions (Fig. 1). The overall GC content was 36.61–36.66% and it was nearly identical in all *Colobanthus* cp genomes (Table 1). All of the analyzed *Colobanthus* cp genomes contained an identical set of 112 genes composed of 73 protein-coding genes, 30 tRNA genes, four rRNA genes and five conserved chloroplast ORFs (*ycf1*, *ycf2, ycf3*, *ycf4*, *ycf68*) (Table 2). Fifty-eight protein-coding genes, 22 tRNA genes and 2 conserved chloroplast ORFs (*ycf3* and *ycf4*) are located in LSC, whereas the SSC region contained 11 protein-coding genes and one tRNA gene. The IR region contained four rRNA genes, seven tRNA genes and eight protein-coding genes, including *ycf2*, *ycf68* and *ycf1* on the border between IR_A_/IR_B_ and SSC. The full *ycf1* sequence is located on the IR_A_/SSC border, and its incomplete copy on the IR_B_/SSC border acts as a pseudogene. Fifteen genes contained one intron (*atpF*, *ndhA*, *ndhB*, *petB*, *petD*, *rpl16*, *rpoC1*, *rps16*, *trnI-*GAU, *trnA*-UGC, *trnK*-UUU, *trnG*-UCC, *trnL*-UAA, *trnV*-UAC, *ycf3*), and two genes consisted of three exons (*rps12* and *clpP*). The first exon of *rps12* (5′ end of the sequence) was found in the LSC region, and the remaining two exons were located in the IR region. This unique feature supported the identification of *rps12* as a trans-spliced gene. The introns of the two remaining genes, *trnK*-UUU and *trnI*-GAU, include coding sequences for *matK* and *ycf68*, respectively.

**Table 1:** Summary of chloroplast genome characteristic of *Colobanthus*.

Genome features	*C. acicularis*	*C. affinis*	*C. lycopodioides*	*C. nivicola*	*C. pulvinatus*	*C. subulatus*
Raw data reads no	5,263,336	4,940 388	5,780,642	1,986 760	5,473,888	7,849,218
Mapped reads no	352,843	224 899	366,573	210,882	233,901	414 160
Percent of chloroplast genome reads (%)	6.70	4.55	6.34	10.61	4.27	5.28
Mean coverage (x)	338.0	216.6	355.5	202.7	220.6	396.5
Size (bp)	151,050	151 315	151,462	151,209	151,145	151,259
LSC length (bp)	83,198	83,419	83,645	83,351	83,306	83,456
SSC length (bp)	17,224	17,256	17,177	17,206	17,188	17,185
IR length (bp)	25,314	25,320	25,320	25,326	25,326	25,309
Number of unique genes	112	112	112	112	112	112
Protein-coding genes	78	78	78	78	78	78
tRNA genes	30	30	30	30	30	30
rRNA genes	4	4	4	4	4	4
Number of genes duplicated in IR	19	19	19	19	19	19
Overall GC content (%)	36.66	36.65	36.61	36.65	36.66	36.66

Species arranged alphabetically.

**Table 2.** Genes present in six chloroplast genomes of *Colobanthus* species.

Category	Group of gene	Name of genes
Photosynthesis	Photosystem I	*psaA*, *psaB*, *psaC*, *psaI*, *psaJ*
	Photosystem II	*psbA*, *psbB*, *psbC*, *psbD*, *psbE*, *psbF*, *psbH*, *psbI*, *psbJ*, *psbK*, *psbL*, *psbM*, *psbN*, *psbT*, *psbZ*
	Cytochrome complex	*petA*, *petB*, *petD*, *petG*, *petL*, *petN*
	ATP synthase	*atpA*, *atpB*, *atpE*, *atpF*, *atpH*, *atpI*
	NADH dehydrogenase	*ndhA*, *ndhB* (×2), *ndhC*, *ndhD*, *ndhE*, *ndhF*, *ndhG*, *ndhH*, *ndhI*, *ndhJ*, *ndhK*
	Large subunit of RUBISCO	*rbcL*
DNA replication and protein synthesis	Ribosomal RNA	*rrn4.5* (×2), *rrn5* (×2), *rrn16* (×2), *rrn23* (×2)
	Small subunit ribosomal proteins	*rps2*, *rps3*, *rps4*, *rps7* (×2), *rps8*, *rps11*, *rps12* (×2), *rps14*, *rps15*, *rps16*, *rps18*, *rps19*^b^
	Large subunit ribosomal proteins	*rpl2* (×2), *rpl14*, *rpl16*, *rpl20*, *rpl22*, *rpl32*, *rpl33*, *rpl36*
	RNA polymerase subunits	*rpoA*, *rpoB*, *rpoC1*, *rpoC2*
	Transfer RNA	*trnA-UGC* (×2), *trnC-GCA*, *trnD-GUC*, *trnE-UUC*, *trnF-GAA*, *trnfM-CAU*, *trnG-GCC*, *trnG-UCC*, *trnH-GUG*, *trnI-CAU* (×2), *trnI-GAU* (×2), *trnK-UUU*, *trnL-CAA* (×4), *trnL-UAA*, *trnL-UAG*, *trnM-CAU*, *trnN-GUU* (×2), *trnP-UGG*, *trnQ-UUG*, *trnR-ACG* (×2), *trnR-UCU*, *trnS-GCU*, *trnS-GGA*, *trnS-UGA*, *trnT-GGU*, *trnT-UGU*, *trnV-GAC* (×2), *trnV-UAC*, *trnW-CCA*, *trnY-GUA*
Other genes	Conserved hypothetical chloroplast ORF	*ycf1*^b^, *ycf2* (×2), *ycf3*^a^, *ycf4*^a^, *ycf68* (×2)
	Other proteins	*accD*, *ccsA*, *cemA*, *clpP*, *matK*

Genes list arranged alphabetically.

^a^Genes associated with Photosystem I.

^b^Gene with its pseudogene copy at IR_B_/LSC and IR_A_/SSC border: *ψrps19* and *ψycf1,* respectively.

The total number of codons for all protein-coding genes in the cp genomes of six *Colobanthus* species ranged from 25,159 to 26,162. The most and least abundant codons (excluding these associated with the initiation and termination of translation) were ATT (4.33%) and TGC (0.25%), respectively (Supplementary Table S1 online). Furthermore, leucine appeared as the dominant amino acid (10.7%), whereas cysteine was less frequently encountered (1.2%). Since the data for codon usage were not available for the previously published plastomes of *C. quitensis* and *C. apetalus*, these species were included in the analysis. Both species shared the same pattern of codon usage and amino acid frequency.

**Figure 2:**
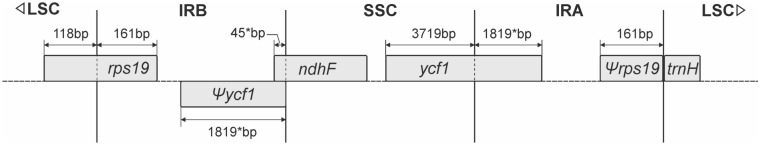
Comparison of LSC, SSC, and IR boundaries of six *Colobanthus* chloroplast genomes. Asterisk represents the location of one base shift in IR_A_/SSC and SSC/IR_B_ boundary position; 45 and 1,819 bp values should be replaced by 44 and 1,818 bp for *C. subulatus.*

The boundaries between IR and SSC/LSC regions in all *Colobanthus* cp genomes were identified (Fig. 2). The IR_A_/SSC junction was found within the *ycf1* gene (1819 bp from its 5′ end), and the boundary between IR_B_ and LSC region was identified within the *rps19* gene (161 bp from its 5′ end). Consequently, the full *ycf1* sequence is located only on the IR_A_/SSC border, and its incomplete copy on IR_B_/SSC border acts as a pseudogene (*ψycf1*). A similar situation was observed for *rps19*, for which the complete sequence can be found on the IR_B_/LSC border, whereas the *ψrps19* pseudogene is located in the IR_A_ region. The IR_B_/SSC boundary was identified within the *ndhF* gene, and a 45 bp string from its 3′-end overlapped *ψycf1* within IR_B_. The IR_A_/LSC junction was adjacent to the *trnH* gene. The boundaries between the IR and SSC/LSC regions of six *Colobanthus* species were generally found in the same positions and within the same genomic elements. One base shift in the IR_A_/SSC and IR_B_/SSC border position was found only in *C. subulatus* (Fig. 2).

### Repetitive sequences and SSRs

**Figure 3:**
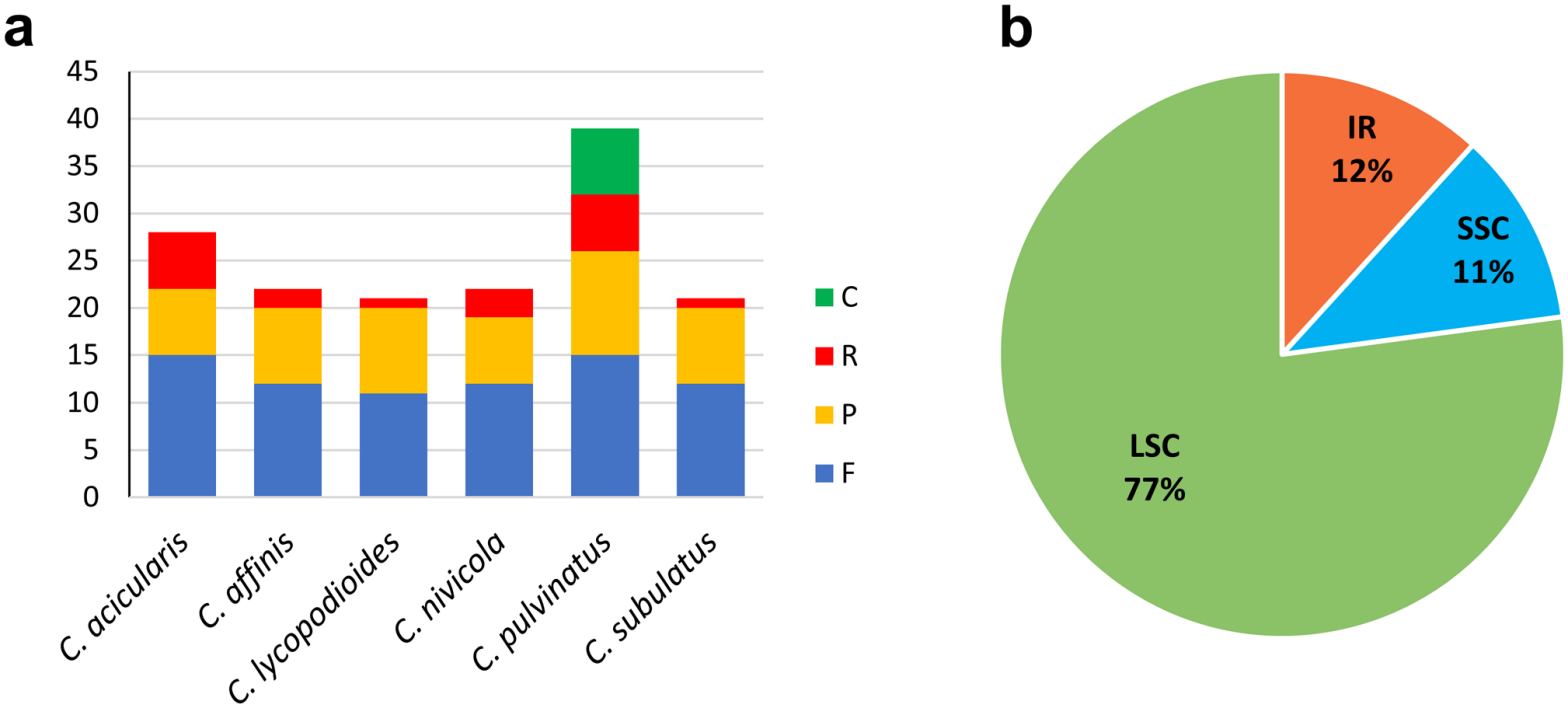
Number of repeat types and distribution of repeats in six *Colobanthus* species. (**A**) Types of repeats, (**B**) Location of repeat sequences. F, P, R and C represent forward, palindromic, reverse and complementary repeats.

A total of 153 repeats were observed in the plastomes of six *Colobanthus* species. The number of repeats was highest (39) in *C. pulvinatus* and lowest (21) in *C. lycopodioides* and *C. subulatus* (Supplementary Table S2A–F online). Forward repeats dominated in the identified repetitive sequences (from 38.5% in *C. pulvinatus* to 57.1% in *C. subulatus*), followed by palindromic (from 25% in *C. acicularis* to 42.9% in *C. lycopodioides*) and reverse repeats (from 4.8% in *C. lycopodioides* and *C. subulatus* to 21.4% in *C. acicularis*). Complementary repeats were found only in the cp genome of *C. pulvinatus* with a frequency of 17.9% (Fig. 3A). Most repeat sequences (77.1%) were detected in the LSC region, followed by IR (11.8%) and SSC regions (11.1%) (Fig. 3B). These sequences were found predominantly within intergenic spacers and introns with a frequency of 79.5% (*C. pulvinatus*) to 57.1% (*C. subulatuc* and *C. lycopodioides*). Our study also revealed that many repeats shared the same locus in all *Colobanthus* cp genomes. Fourteen such loci were identified: *psaA*, *psaA–ycf3*, *psaB*, *psaI–ycf4*, *petN–psbM*, *trnG-GCC*, *trnG-UCC*, *trnS-GCU*, *trnS-GGA* and *trnS-UGA* in the LSC region, *ndhA* and *ycf1* in the SSC region, and *trnV-GAC-rps7* and *ycf2* in the IR region.

**Figure 4:**
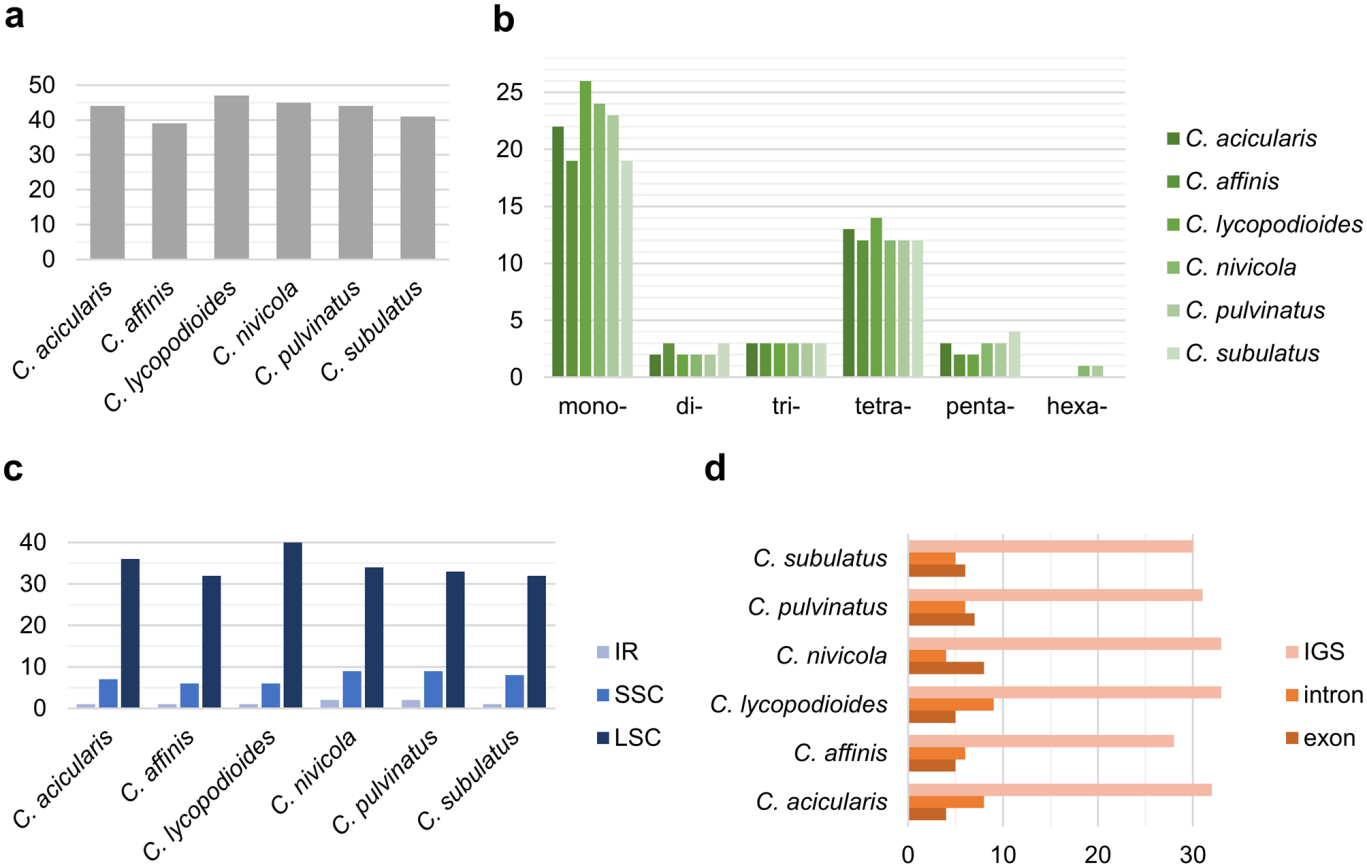
The distribution and type of simple sequence repeats (SSRs) in cp genomes of six *Colobanthus* species. (**A**) Number of different SSRs types. (**B**) Location of different SSRs in IR, SSC and LSC regions. (**C**) Distribution of SSR motifs in different repeat class types. (**D**) Partition of SSRs among IGS, introns and exons.

The Phobos analysis supported the identification of 39–47 SSRs in six cp genomes (Fig. 4A), including mono-, di-, tri-, tetra-, penta- and hexanucleotides (Supplementary Table S3A–F online). The mononucleotide SSRs were most common with a frequency ranging from 46.3% in *C. subulatus* to 55.3% in *C. lycopodioides* (Fig. 4B). All mononucleotide SSRs were composed of A/T repeat units. Motifs composed of adenine and thymine were also predominant in di- and trinucleotide SSRs, where only AT/TA and AAT/TTA motifs were observed, respectively. Tetranucleotide SSRs were the second most frequent repeats that ranged from 26.7% in *C. nivicola* to 30.8% in *C. affinis*. The frequency of di-, tri- and pentanucleotide SSRs did not exceed 9.8%. Hexanucleotide SSRs were detected only in *C. nivicola* and *C. pulvinatus* with a frequency of 2.2% and 2.3%, respectively. The majority of SSRs were located in the LSC region (from 75% in *C. pulvinatus* to 85.1% in *C. lycopodioides*), followed by SSC (from 12.8% in *C. lycopodioides* to 20.5% in *C. pulvinatus*) and IR regions (from 2.1% in *C. lycopodioides* to 4.5% in *C. pulvinatus*) (Fig. 4C). Furthermore, SSRs were identified predominantly within intergenic spacers (from 70.2% in *C. lycopodioides* to 73.3% in *C. nivicola*), whereas the remaining SSRs were distributed in various proportions between exons and introns (Fig. 4D).

### Sequence divergence

**Figure 5:**
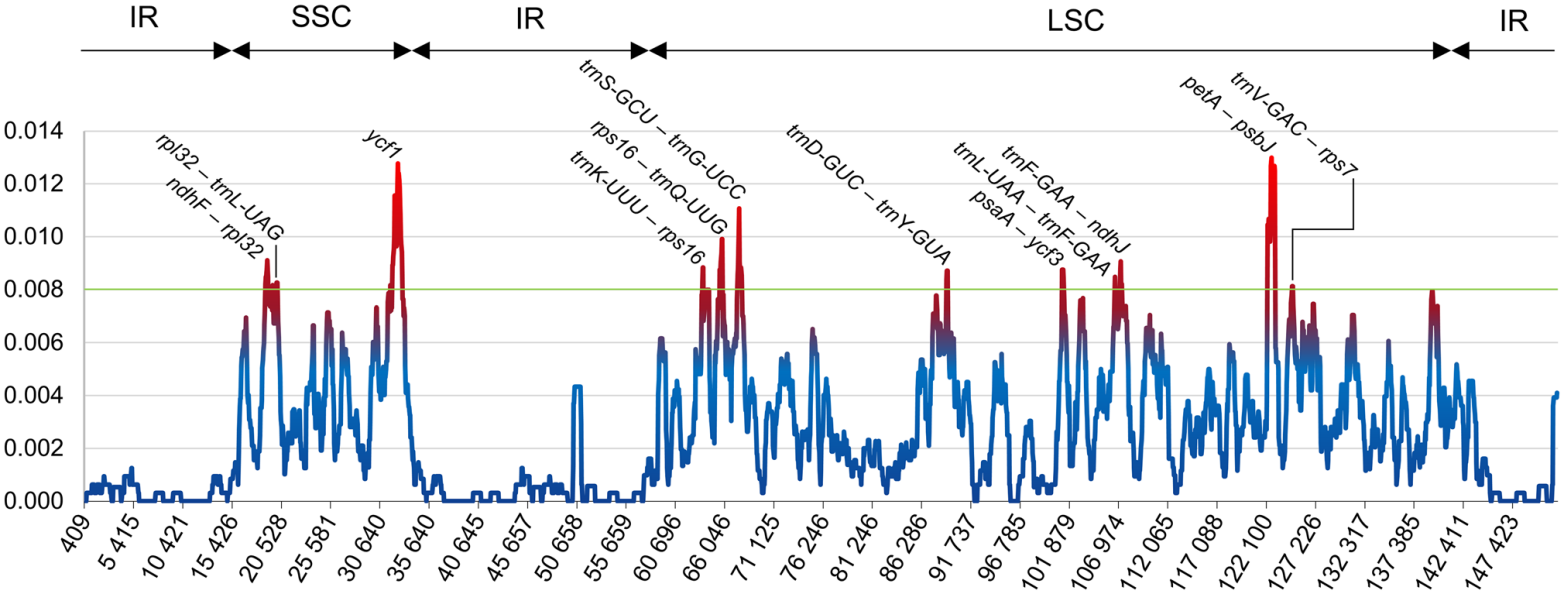
Sliding window analysis of the eight *Colobanthus* complete chloroplast genome sequences (window length: 800 bp; step size 50 bp). The Y-axis presents nucleotide diversity of each window, while the X-axis represents position of the midpoint.

The overall sequence identity and divergent regions in the cp genomes of *C. acicularis*, *C. affinis*, *C. lycopodioides*, *C. nivicola*, *C. pulvinatus*, *C. subulatus* and the previously published plastomes of *C. quitensis* and *C. apetalus*^25,26^ were determined in MAUVE and DnaSP programs. MAUVE results are shown in Supplementary Figure S1 online. Rearrangements (inversions or translocations) were not detected in any of the eight chloroplast genome sequences. The high sequence similarity points to the conservative character of all eight cp genomes. In the DnaSP, nucleotide diversity (π) in the cp genomes of *Colobanthus* species was determined at 0.00262. The most variable regions were identified in sliding window analysis, i.e. regions for which π values exceeded 0.008 (Fig. 5). Divergence was generally higher in non-coding regions. In the coding region, differences were found only in the *ycf1* locus. In non-coding regions, the highest divergence and the highest π value (0.01299) were observed for *ndhF–rpl32*, *rpl32–trnL-UAG*, *trnK-UUU–rps16*, *rps16–trnQ-UUG*, *trnS-GCU–trnG-UCC*, *trnD-GUC–trnY-GUA*, *psaA–ycf3*, *trnL-UAA–trnF-GAA*, *trnF-GAA–ndhJ*, *petA–psbJ* and *trnV-GAC–rps7* (Supplementary Table S4 online). The majority of highly variable regions (9) were identified in LSC. There were three such regions in SSC, and none in the IR region.

### Synonymous (Ks) and non-synonymous (Ka) substitution rate analysis

The substitution rate varied widely across plastome genes in each functional group, and the values of Ka and Ks were determined in the range of 0–0.0117 and 0–0.152, respectively (Supplementary Table S5 online). The highest average value of Ks (0.0111) was noted for coding sequences associated with the large subunit of ribosome. The average value of Ks was lowest in genes related to the cytochrome b/f complex (0.0008), RubisCO large subunit (0.0015), Photosystem II (0.0022) and Photosystem I (0.0026). The genes associated with Photosystem II, Photosystem I and the cytochrome b/f complex were also characterized by the lowest average values of Ka (0.0003, 0.0005 and 0.0005, respectively), and the highest average value of Ka (0.0025) was noted in the RubisCO large subunit. In general, no differences were observed in the sequences of 18 plastome genes (Ka = 0, Ks = 0) of the studied *Colobanthus* species. The remaining 60 genes shared 99% similarity, but only synonymous substitutions (Ka = 0) were observed in 33 of those genes. The Ka/Ks ratio was less than 1 in all genes, excluding *rpoC2* (1.5238 for *C. affinis*, and 1.381 for *C. nivicola* and *C. pulvinatus*) and *matK* (1.1333 for *C. lycopodioides*). The Ka/Ks ratio exceeded 1 in *rpoC2* and *matK,* which could suggest that in the above species, these genes had undergone positive selection (adaptation to a specific environment). A Ka/Ks ratio of less than 1 points to the influence of purifying selection on the remaining 76 genes.

### RNA-editing

The results of the PREP prediction revealed 49 editing sites in 18 protein coding genes in the plastomes of *Colobanthus* species, excluding *C. acicularis* and *C. lycopodioides* where 48 such elements were found (Supplementary Table S6 online). One RNA editing site within the *ndhF* gene was missing in *C. acicularis*, and one editing site within the *ndhB* sequence was missing in *C. lycopodioides*. All editing events involved C to U conversion. Fifteen non-synonymous mutations were found at the first position of the codon, 34 mutations were identified at the second position, and none were found at the third position. Serine (S) to leucine (L) changes accounted for nearly a third (32.6%) of the identified mutations, whereas arginine (R) to tryptophan (W) and serine (S) to phenylalanine (F) changes were least frequently observed (4.1% for both). Each RNA editing site in the corresponding genes of the eight *Colobanthus* species was generally found at the same nucleotide position. Three base shifts were identified in only two RNA editing sites within the *rpoB* sequence in the cp genome of *C. quitensis*.

### Phylogenetic analysis

**Figure 6:**
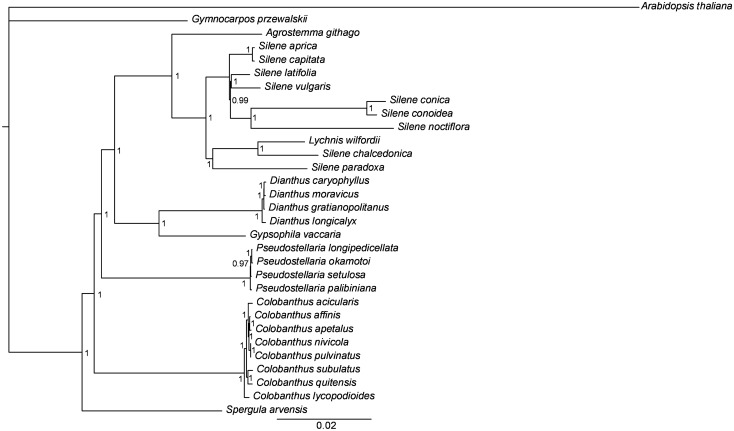
Phylogenetic tree based on sequences of sheared 73 protein-coding genes from eight *Colobanthus* species and 22 other Caryophyllaceae representatives using Bayesian posterior probabilities (PP). Bayesian PP are given at each node.

The phylogenetic trees generated by BI and ML had a consistent topology. In the BI tree, Bayesian posterior probability reached 1.0 in 92.6% of the nodes (25 out of 27). The reconstructed phylogeny is consistent with the taxonomic position of the studied species, and it revealed the following relationships: all studied *Colobanthus* species formed one clade; the second clade grouped four *Pseudostellaria* species; the third clade consisted of four *Dianthus* species and a solitary branch of *Gysophilla vacaria*; all members of the genus *Silene* and *Lychnis wilfordii* formed the fourth clade in the proximity of a separate branch of *Agrostemma githago*; the most divergent branches were formed by *Gymnocarpos przewalski* and *Spergula arvensis* (Fig. 6, Table 3).

**Table 3:** GenBank accession numbers and references for cp genomes used in this study.

Species	Accession number	Length (bp)	Reference
*Agrostemma githago*	NC_023357	151,733	Sloan et al.^54^
*Arabidopsis thaliana*	NC_000932	154,478	Sato et al.^103^
*Colobanthus acicularis*	MN273320	151,050	This study
*Colobanthus affinis*	MN273318	151,315	This study
*Colobanthus apetalus*	NC_036424	151,228	Androsiuk et al.^26^
*Colobanthus lycopodioides*	MN273317	151,462	This study
*Colobanthus nivicola*	MN273316	151,209	This study
*Colobanthus pulvinatus*	MN273315	151,145	This study
*Colobanthus subulatus*	MN273319	151,259	This study
*Colobanthus quitensis*	NC_028080	151,276	Kang et al.^25^
*Dianthus caryophyllus*	NC_039650	147,604	Chen et al. (2018)^a^
*Dianthus gratianopolitanus*	LN877389	149,735	Michling et al. (2018)^a^
*Dianthus longicalyx*	KM668208	149,539	Gurusamy et al.^104^
*Dianthus moravicus*	LN877396	149,524	Michling et al. (2018)^a^
*Gymnocarpos przewalskii*	NC_036812	150,636	Yang (2017)^a^
*Gypsophila vaccaria*	NC_040936	150,042	Yao et al.^105^
*Pseudostellaria longipedicellata*	NC_039454	149,626	Kim et al. (2018)^a^
*Pseudostellaria okamotoi*	NC_039974	149,653	Kim et al. (2019)^a^
*Pseudostellaria palibiniana*	NC_041166	149,668	Kim and Park (2019)^a^
*Pseudostellaria setulosa*	MK172842	149,479	Kim and Park (2019)^a^
*Lychnis wilfordii*	NC_035225	152,320	Kang et al.^69^
*Silene aprica*	NC_040934	150,293	Yao et al.^105^
*Silene capitata*	NC_035226	150,224	Kang et al.^69^
*Silene chalcedonica*	NC_023359	148,081	Sloan et al.^54^
*Silene conica*	NC_016729	147,208	Sloan et al.^53^
*Silene conoidea*	NC_023358	147,896	Sloan et al.^54^
*Silene latifolia*	NC_016730	151,736	Sloan et al.^53^
*Silene noctiflora*	NC_016728	151,639	Sloan et al.^53^
*Silene paradoxa*	NC_023360	151,632	Sloan et al.^54^
*Silene vulgaris*	NC_016727	151,583	Sloan et al.^53^
*Spergula arvensis*	NC_041240	152,703	Yao et al.^105^

Species list arranged alphabetically.

^a^Direct submission to NCBI, unpublished.

## Discussion

The length of the complete sequences of the six new chloroplast genomes of *Colobanthus* species ranged from 151,050 (*C. acicularis*) to 151,462 bp (*C. lycopodioides*), it was very similar to the previously sequenced plastomes of *C. quitensis* (151,276 bp)^25^ and *C. apetalus* (151,228 bp)^26^, and was within the size range of cp genomes of other angiosperms^45^. A comparison of all *Colobanthus* cp genomes that have been sequenced to date also revealed considerable similarities in genome composition—all eight species had the same gene content and order. Moreover, their protein-coding sequences were characterized by low variation. Consequently, the differences in the size and organization of intergenic spacers were most probably responsible for the observed variations in the size of *Colobanthus* cp genomes. As previously described^46^, the variation in the size of cp genomes in different plant lineages could also be attributed to the expansion and contraction of IR regions. Similarly to most angiosperms, IR boundaries were found within *ycf1* and *rps19* genes in the presented cp genomes of *Colobanthus*^47^. The location of IR boundaries was identical in all *Colobanthus* species. One base shift in the position of IR_A_/SSC and IR_B_/SSC borders was found only in *C. subulatus*. The size of IR regions was highly similar in all *Colobanthus* cp genomes that have been sequenced to date, ranging from 25,303 (*C. quitensis*) to 25,326 bp (*C. nivicola* and *C. pulvinatus*), which corresponds to the values reported in other dicotyledons^48, 49^. Somewhat greater differences were observed in *Colobanthus* species when the length of LSC and SSC regions was considered. The differences in the length of LSC and SSC between the longest and the shortest element were determined at 447 bp (*C. lycopodioides* vs. *C. acicularis*) and 79 bp (*C. affinis* vs. *C. lycopodioides*), respectively.

The accumulation of point mutations in the form of synonymous and non-synonymous nucleotide substitutions is one of the key mechanisms of gene evolution^50^. In this study, synonymous nucleotide substitutions were more frequently observed; therefore, the analyzed *Colobanthus* species have maintained a high degree of sequence conservation, especially in genes involved in photosynthesis. However, considerable variation was observed in several genes, including *rps16* and *ycf1* which are characterized by the highest number of non-synonymous nucleotide substitutions. These two elements of chloroplast genomes are very often found among the most variable chloroplast loci of numerous genera^51^, including *Silene* (Caryophyllaceae)^52–54^. The Ka/Ks ratio was examined to determine selection pressure on protein-coding genes. The six *Colobanthus* cp genomes exhibited highly conserved organization, but positive selection pressure (Ka/Ks > 1) was observed in *rpoC2* (*C. affinis*, *C. nivicola* and *C. pulvinatus*) and *matK* (*C. lycopodioides*), which suggests that these loci are undergoing essential adaptation to environmental conditions. In *C. lycopodioides*, *C. nivicola* and *C. pulvinatus,* the Ka/Ks ratio for the *rbcL* gene was only somewhat lower than 1 (0.965), which could indicate that positive selection played some role in the acceleration of the substitution rate for that locus. All three genes have been also previously reported to undergo positive selection in other plant species. Gene *rbcL*, which encodes the large subunit of RuBisCO, appears to undergo positive selection most often, in up to 75–88% of terrestrial plants^55^. According to the literature, *rbcL* could be a chloroplast region that was positively selected during the evolutionary processes associated with adaptation to temperature^55–58^, CO_2_ concentration^55,59,60^ or water deficiency^57,60^. The *matK* gene encoding the maturase enzyme which catalyzes the removal of a nonautocatalytic intron from premature RNAs^61^ was also found to undergo positive selection in 32 plant groups^62^. Despite its important function, the *rpoC2* gene encoding the β subunit of plastid-encoded plastid RNA polymerase (RNA polymerase type I) is also a relatively rapidly evolving chloroplast sequence^63^ that undergoes positive selection in various groups of plants, including Lamiaceae^64^, Orobanchaceae^65^ and Annonaceae^66^. The fact that traces of positive selection were detected in such different functional gene classes, including genes encoding photosynthesis (*rbcL*), transcription and transcript processing (*matK* and *rpoC2*), could indicate that natural selection targets different chloroplast functions. A higher rate of substitutions in these *Colobanthus* genes could be indicative of continuous fine-tuning to specific environmental conditions.

Repeat regions within genomes play an important role in sequence divergence and rearrangement, which is why they have to be identified, and their number and distribution has to be determined in genomic studies^67,68^. In the six reported here plastomes of *Colobanthus* species, most repeat regions were identified in intergenic regions and introns (57.1–79.5%), which is highly consistent with the values previously reported in *C. apetalus* (76.7%) and *C. quitensis* (53.3%)^26^, as well as for other Caryophyllaceae such as *Silene capitata* (56.0%) and *Lychnis wilfordii* (69.2%)^69^. Our study also demonstrated that repeat regions are not randomly distributed within *Colobanthus* cp genomes, and they were identified mainly within highly divergent regions of *rpl32–trnL-UAG*, *ycf1*, *trnK-UUU–rps16*, *psaA–ycf3* and *petA–psbJ* in the LSC.

Simple sequence repeats, also known as microsatellites, are important molecular markers with many applications, including species identification, population genetics and phylogenetic studies^70–72^. Mononucleotide SSRs were identified most frequently (51% on average) among the microsatellites of the six analyzed cp genomes of *Colobanthus* species, with A/T as the prevalent motif type. The frequency of mononucleotide SSRs was highly similar in *C. apetalus* and *C. quitensis* (48.8% and 54.2%, respectively)^26^ as well as in other plant species, including members of the family Caryophyllaceae^69,73,74^. In turn, hexanucleotide SSRs were least abundant, and only one such element was identified in the cp genomes of *C. nivicola* and *C. pulvinatus*. Moreover, the majority of SSRs were located within intergenic spacers and introns, while only 13.5% (on average) were positioned in the exons of 11 genes. Four of these loci (*ycf1*, *rpoC2, rrn23*, *atpA*) harbored SSRs in all six *Colobanthus* species, whereas four other loci (*petB*, *petD*, *ndhA*, *rpoA*) contained SSRs in only one, specific species.

In terrestrial plants, cytidines are systematically converted to uridines (C to U editing) in both mitochondrial and plastid mRNA transcripts to restore conserved codons^75^. This important post-transcriptional process that had appeared in early stages of flowering plant evolution^76^ is considered to be functionally significant in chloroplasts^77^. In our work, potential RNA editing sites were identified in 18 out of the 34 analyzed chloroplast protein-coding genes. These sites were highly conserved in the analyzed species, excluding two sites within the *rpoB* gene in the cp genome of *C. quitensis*. In this species, the three base shift appear to be a consequence of the insertion of three bases (CAG) which added glutamine in position 636 of the *rpoB* amino acid sequence. This study also demonstrated that leucine codons, including those that have potentially emerged from RNA editing, are heavily used in the cp genomes of all eight *Colobanthus* species. Previous research revealed a high demand for leucine biosynthesis in chloroplasts and suggested that this amino acid plays an important role in photosynthesis-related metabolism^78,79^.

*Colobanthus* is one of the 86 genera within the family Caryophyllaceae^42^. Genus *Colobanthus* contains around 26 confirmed species and 24 taxa whose status has not been resolved^1^. Therefore, a taxonomic inventory of the genus *Colobanthus* is still needed. However, this is a challenging task due to the extensive geographic distribution of the species and high variation within morphological traits. The DNA barcode has recently emerged as an effective biological tool for accurate species identification^80^. Despite the above, the molecular data for *Colobanthus* are highly limited, and species-specific barcode sequences are not available. In the eight *Colobanthus* species with completely sequenced cp genomes, the genetic variation in the chloroplast regions of *matK* and *rbcL* that are widely used in the barcoding of terrestrial plants was lower than expected. However, genome-wide comparative analyses based on nucleotide diversity (π) supported the identification of 12 highly variable regions (π > 0.008) that could be utilized as a source of potential markers for species identification and reconstruction of the phylogenetic relationships within this plant group: *ndhF–rpl32*, *rpl32–trnL-UAG*, *ycf1*, *trnK-UUU–rps16*, *rps16–trnQ-UUG*, *trnS-GCU–trnG-UCC*, *trnD-GUC–trnY-GUA*, *psaA–ycf3*, *trnL-UAA–trnF-GAA*, *trnF-GAA–ndhJ*, *petA–psbJ* and *trnV-GAC–rps7*. High nucleotide diversity (π) in regions *ycf1*, *rpl32–trnL*, *trnS–trnG*, *petA–psbJ* and *rps16–trnQ* was also reported in other plants^51^. Moreover, the *ndhF–rpl32–trnL-UAG* region has been widely used in phylogenetic studies^81,82^. Two of these highly variable regions (*rps16–trnQ-UUG* and *trnL-UAA–trnF-GAA*) were among the 5 chloroplast sequences that were analyzed by Greenberg and Donoghue^83^ to explore the phylogenetic relationships within the family Caryophyllaceae.

It has recently been postulated that complete chloroplast genome sequences can be used as a super barcode for identifying plant species^84^. This approach is particularly useful for distinguishing between closely related taxa where limited sequence variation has resulted from a low rate of genome evolution or a relatively short time since the divergence event^85,86^. Considering the still unresolved status of many *Colobanthus* species, a phylogeny reconstruction based on whole plastome sequences seems to be highly desired for this genus. In this study, chloroplast genome sequences were used to resolve the phylogenetic relationships within the genus *Colobanthus* and the Caryophyllaceae family, but the analysis involved only species with completely elucidated plastome sequences. The phylogeny reconstructed based on 73 concatenated protein-coding gene sequences appeared to be consistent with the taxonomic position of the studied species and previous phylogenies of the Caryophyllaceae^41,83,87^. However, in the genus *Colobanthus,* this was an initial step towards resolving the phylogenetic relationships within that group of plants. The phylogenetic tree with highly supported nodes revealed that all eight *Colobanthus* species were grouped in one clade, where *C. pulvinatus* and *C. nivicola*, *C. apetalus* and *C. affinis* as well as *C. subulatus* and *C. quitensis* formed three pairs of most similar species, whereas *C. lycopodioides* appeared to be most different.

## Conclusion

The chloroplast genomes of *C. acicularis*, *C. affinis*, *C lycopodioides*, *C. nivicola*, *C. pulvinatus* and *C. subulatus* were sequenced and characterized for the first time. Their plastomes have a typical quadripartite circular structure and share the same overall organization and gene content. The information regarding sequence variation, distribution and characteristics of SSR loci within the studied cp genomes could be useful in future studies on the population genetics, phylogenetics and evolution of *Colobanthus*. Nevertheless, further research is required to investigate whether highly variable regions or complete chloroplast genome sequences could be used as reliable and effective DNA barcodes for *Colobanthus* species*.*

## Methods

### Plant material, DNA extraction and chloroplast genome sequencing

Fresh leaves of five *Colobanthus* species were sampled from plants grown from seeds in a greenhouse of the Department of Plant Physiology, Genetics and Biotechnology at the University of Warmia and Mazury in Olsztyn, Poland. The seeds of *C. affinis* were obtained from the Royal Botanic Gardens, Victoria, Australia. The seeds of *C nivicola* and *C. pulvinatus* were acquired from the Australian National Botanic Gardens, Canberra. The seeds of *C. subulatus* originated from the Royal Botanic Gardens, Kew, United Kingdom. The seeds of *C. lycopodioides* were collected in the region of Mendoza, Andes, Argentina, at an altitude of 4,024 m a.s.l., (33°10′ S; 69°50′ W). Only in *C. acicularis,* DNA was extracted from dried tissue of one individual, supplied in silica gel by the Royal Botanic Garden, Edinburgh, UK. Plant material was formally identified before the analyses. Professor Irena Giełwanowska performed morphological and anatomical analyses of both vegetative and generative organs harboring characteristic traits for the identification of *Colobanthus* species^1,88,89^. Voucher specimens of each studied species have been deposited in the Vascular Plants Herbarium of the Department of Botany and Nature Protection at the University of Warmia and Mazury in Olsztyn, Poland (OLS), under the following numbers: *C. acicularis* (No. OLS 33824), *C. affinis* (No. OLS 33825), *C lycopodioides* (No. OLS 33826), *C. nivicola* (No. OLS 33827), *C. pulvinatus* (No. OLS 33828) and *C. subulatus* (No. OLS 33829). Total genomic DNA was extracted from fresh/dry tissue of a single plant using the Maxwell16LEV Plant DNA Kit (Promega, Madison, WI). The quality of DNA was verified on 1% (w/v) agarose gel stained with 0.5 µg/ml ethidium bromide. The concentration and purity of DNA samples were assessed spectrophotometrically.

Genome libraries were prepared using the Nextera XT kit (Illumina Inc., San Diego, CA, USA), and genomes were sequenced on the Illumina MiSeq platform (Illumina Inc., San Diego, CA, USA) with a 150 bp paired-end read.

### Annotation and genome analysis

The FastQC tool was used to check the quality of raw reads. Raw reads were trimmed (5 bp of each read end, regions with more than 5% probability of error per base) and mapped to the reference chloroplast genome of *C. quitensis* (NC_028080) in Geneious v.R7 software^90^ with default medium–low sensitivity settings. Subsequent steps of annotation and genome analysis were described in our previous paper^26^. The gene maps of the annotated cp genomes were developed with the OrganellarGenome DRAW tool^91^.

### Genomic repeats and SSR analysis

The size and location of genomic repeats, including forward, reverse, palindromic and complementary sequences within the analyzed chloroplast genomes, were identified using REPuter software^92^ with the following settings: (1) hamming distance of 3, (2) sequence identity ≥ 90%, and (3) minimum repeat size ≥ 30 bp. Phobos v.3.3.12^93^ was used to detect chloroplast simple sequence repeats (SSRs). Only perfect SSRs with a motif size of one to six nucleotide units were considered, with standard thresholds for chloroplast SSRs identification: ≥ 12 repeat units for mononucleotide SSRs, ≥ 6 repeat units for dinucleotide SSRs, ≥ 4 repeat units for trinucleotide SSRs, and ≥ 3 repeat units for tetra-, penta- and hexanucleotide SSRs^94^. A single IR region was used to eliminate the influence of doubled IR regions. Redundant results in REPuter were deleted manually.

### Comparative chloroplast genome analysis

Genome synteny analysis of the eight *Colobanthus* plastomes (six genomes reported in this paper, and *C. quitensis* and *C. apetalus* that were previously characterized and deposited in NCBI^25,26^) was performed with the use of MAUVE v.1.1.1^95^. The sequences were aligned in MAFFT v.7.310^96^ to perform sliding window analysis and evaluate nucleotide diversity (π) in cp genomes using DnaSP v.6.10.04^97^. The step size was set to 50 base pairs, and window length was set to 800 base pairs.

The evolutionary rate of the plastome genes identified in all *Colobanthus* species (*C. acicularis*, *C. affinis*, *C. apetalus*, *C lycopodioides*, *C. nivicola*, *C. pulvinatus*, *C. quitensis* and *C. subulatus*) was analyzed. A total of 78 genes were selected to estimate the ratio of non-synonymous (Ka) to synonymous (Ks) substitutions. *Colobanthus quitensis* was the reference species. These genes were extracted and aligned separately using MAFFT v7.310. The values of Ka and Ks in the shared genes were calculated in DnaSP v.6.10.04. Genes with non-applicable (NA) Ka/Ks ratios were changed to zero.

The chloroplast genome borders of LSC, SSC, and IRs were identified and compared based on their annotations. The data on the distribution of codon usage was acquired from the Geneious v.7 statistics panel.

Potential RNA editing sites in the protein-coding genes of chloroplast genomes were predicted using the Predictive RNA Editor for Plants (PREP) suite^98^. The cutoff value for the analyzed *Colobanthus* species was set at 0.8, and 34 out of the 35 reference genes in PREP were used. *rpl23* was not included in the analysis because it was not identified within the chloroplast genomes of the studied *Colobanthus* species*.* Two previously sequenced cp genomes of *C. quitensis* and *C. apetalus*^25,26^ were also included in this analysis.

### Phylogenetic analysis

The available (24) complete chloroplast genomes representing Caryophyllaceae lineages and the cp genome of *Arabidopsis thaliana* as an outgroup were downloaded from the NCBI database to investigate the phylogenetic relationships among the studied representatives of the genus *Colobanthus* and the genera in the family Caryophyllaceae. The cp genomes used in phylogenetic analyses are presented in Table 3. The sequences of 73 shared protein coding genes were extracted using custom R script, and they were aligned in MAFFT v.7.310. Finally, 73 concatenated protein-coding gene sequences where used for phylogeny reconstruction by Bayesian Inference (BI) and Maximum-Likelihood (ML) method. The best-fit model of sequence evolution was identified in MEGA v.7^99^, and the GTR + G + I model was selected. The BI analysis was performed in MrBayes v.3.2.6^100,101^, and the ML analysis was conducted in PhyML v.3.0^102^. Parameter settings were previously described by Androsiuk et al.^26^.

## Supplementary information

Supplementary Figure S1.

Supplementary Table S1.

Supplementary Table S2.

Supplementary Table S3.

Supplementary Table S4.

Supplementary Table S5.

Supplementary Table S6.

## Data Availability

The complete chloroplast genomes of the six *Colobanthus* species have been submitted to the NCBI database (https://www.ncbi.nlm.nih.gov/https://www.ncbi.nlm.nih.gov/) under the accession numbers: MN273320 for *C. acicularis*, MN273318 for *C. affinis*, MN273317 for *C. lycopodioides*, MN273316 for *C. nivicola*, MN273315 for *C. pulvinatus* and MN273319 for *C. subulatus*.
