# Supplementary material for: Evolutionary dynamics of the chloroplast genome sequences of six Colobanthus species
Source: Sci Rep. 2020 Jul 13;10:11522. doi: 10.1038/s41598-020-68563-5 (PMC7359349; doi:10.1038/s41598-020-68563-5)
Supplement: Supplementary file 1 — Supplementary Figure S1. [file 41598_2020_68563_MOESM1_ESM.pdf]

Evolutionary dynamics of the chloroplast genome sequences of six *Colobanthus* species

Piotr Androsiuk*[1], Jan Paweł Jastrzębski[1], Łukasz Paukszto[1], Karol Makowczenko[1], Adam Okorski[2], Agnieszka Pszczółkowska[2], Katarzyna Joanna Chwedorzewska[3], Ryszard Górecki[1], Irena Giełwanowska[1]

1. Department of Plant Physiology, Genetics and Biotechnology, University of Warmia and Mazury in Olsztyn, ul. M. Oczapowskiego 1A, 10-719 Olsztyn, Poland.
2 Department of Entomology, Phytopathology and Molecular Diagnostics, University of Warmia and Mazury in Olsztyn, ul. Prawocheńskiego 17, 10-720 Olsztyn, Poland.
3 Department of Agronomy, Warsaw University of Life Sciences-SGGW, ul. Nowoursynowska 166, 02-787 Warszawa, Poland.

Figure S1. A MAUVE alignment of *C. acicularis, C. affinis*, *C. apetalus. C. lycopodioides*, *C. nivicola*, *C. pulvinatus*, *C. subulatus* and *C. quitensis* chloroplast genomes showing the lack of rearrangements between the chloroplast genomes of the eight species. The *C. quitensis* genome is shown at top as the reference. Within each of the alignment, local collinear blocks are represented by blocks of the same color connected by lines.

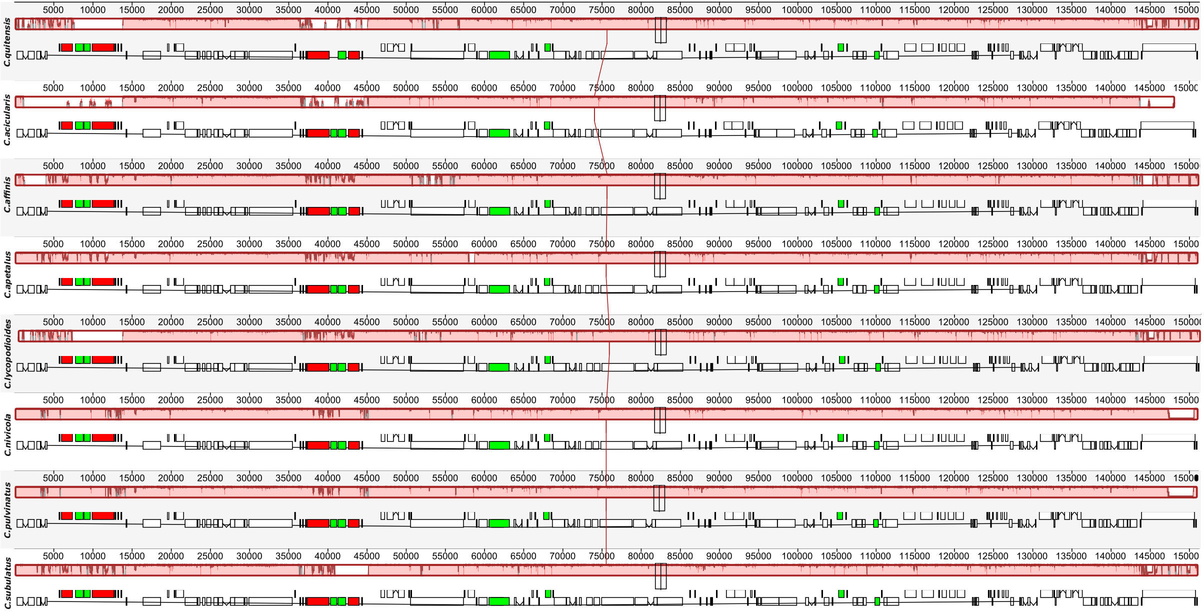
C.quitensis
C.acicularis
C.affinis
C.apetalus
C.lycopodioides
C.nivicola
C.pulvinatus
C.subulatus
5000 10000 15000 20000 25000 30000 35000 40000 45000 50000 55000 60000 65000 70000 75000 80000 85000 90000 95000 100000 105000 110000 115000 120000 125000 130000 135000 140000 145000
