# Supplementary material for: Evolutionary dynamics of the chloroplast genome sequences of six Colobanthus species
Source: Sci Rep. 2020 Jul 13;10:11522. doi: 10.1038/s41598-020-68563-5 (PMC7359349; doi:10.1038/s41598-020-68563-5)
Supplement: Supplementary file 3 — Supplementary Table S2. [file 41598_2020_68563_MOESM3_ESM.pdf]

Evolutionary dynamics of the chloroplast genome sequences of six *Colobanthus* species

Piotr Androsiuk*[1], Jan Paweł Jastrzębski[1], Łukasz Paukszto[1], Karol Makowczenko[1], Adam Okorski[2], Agnieszka Pszczółkowska[2], Katarzyna Joanna Chwedorzewska[3], Ryszard Górecki[1], Irena Giełwanowska[1]

1. Department of Plant Physiology, Genetics and Biotechnology, Faculty of Biology and Biotechnology, University of Warmia and Mazury in Olsztyn, ul. M. Oczapowskiego 1A, 10-719 Olsztyn, Poland.
2 Department of Entomology, Phytopathology and Molecular Diagnostics, Faculty of Environmental Management and Agriculture, University of Warmia and Mazury in Olsztyn, ul. Prawocheńskiego 17, 10-720 Olsztyn, Poland.
3 Department of Agronomy, Warsaw University of Life Sciences-SGGW, ul. Nowoursynowska 166, 02-787 Warszawa, Poland.

Table. S2A. List of repeated sequences in the chloroplast genome of *Colobanthus acicularis*

Repeat lenght (bp)	Strat site of repeat A	Repeat A location	Repeat A region	Strat site of repeat B	Repeat B location	Repeat B region	Repeat type
30	23989	IGS (*psaC-ndhE*)	SSC	24014	*ndhE*	SSC	F
30	52308	*ycf2*	IR	52350	*ycf2*	IR	F
30	52310	*ycf2*	IR	52328	*ycf2*	IR	F
30	66785	*trnS-GCU*	LSC	93269	*trnS-UGA*	LSC	F
30	66785	*trnS-GCU*	LSC	102808	*trnS-GGA*	LSC	P
30	87815	IGS (*psbM-trnD-GUC*)	LSC	89204	IGS (*trnE-UUC-trnT-GGU*)	LSC	F
30	87816	IGS (*psbM-trnD-GUC*)	LSC	87819	IGS (*psbM-trnD-GUC*)	LSC	F
30	87818	IGS (*psbM-trnD-GUC*)	LSC	87818	IGS (*psbM-trnD-GUC*)	LSC	R
30	87818	IGS (*psbM-trnD-GUC*)	LSC	87819	IGS (*psbM-trnD-GUC*)	LSC	F
30	87818	IGS (*psbM-trnD-GUC*)	LSC	129669	*clpP* (intron1)	LSC	R
30	87818	IGS (*psbM-trnD-GUC*)	LSC	129669	*clpP* (intron1)	LSC	F
30	87819	IGS (*psbM-trnD-GUC*)	LSC	87819	IGS (*psbM-trnD-GUC*)	LSC	R
30	93269	*trnS-UGA*	LSC	102808	*trnS-GGA*	LSC	P
30	96444	*psaB*	LSC	98668	*psaA*	LSC	F
30	100169	IGS (*psaA-ycf3*)	LSC	118169	*ycf4*	LSC	P
30	100309	IGS (*psaA-ycf3*)	LSC	118030	IGS (*psaI-ycf4*)	LSC	P
31	52331	*ycf2*	IR	52355	*ycf2*	IR	F
31	68397	*trnG-UCC*	LSC	94203	*trnG-GCC*	LSC	F
31	87815	IGS (*psbM-trnD-GUC*)	LSC	129668	*clpP* (intron1)	LSC	F
31	87816	IGS (*psbM-trnD-GUC*)	LSC	87818	IGS (*psbM-trnD-GUC*)	LSC	F
31	87818	IGS (*psbM-trnD-GUC*)	LSC	87818	IGS (*psbM-trnD-GUC*)	LSC	R
31	87819	IGS (*psbM-trnD-GUC*)	LSC	129668	*clpP* (intron1)	LSC	R
31	87819	IGS (*psbM-trnD-GUC*)	LSC	129669	*clpP* (intron1)	LSC	F
31	89203	IGS (*trnE-UUC-trnT-GGU*)	LSC	129667	*clpP* (intron1)	LSC	F
38	87187	IGS (*petN-psbM*)	LSC	87187	IGS (*petN-psbM*)	LSC	P
40	26624	*ndhA* (intron)	SSC	45872	IGS (*trnV-GAC-rps7*)	IR	P
41	33205	*ycf1*	SSC	33205	*ycf1*	SSC	R
141	100198	IGS (*psaA-ycf3*)	LSC	118030	IGS (*psaI-ycf4*)	LSC	P

IGS (*psaC-ndhE*) means spacer between *psaC* and *ndhE*, P means palindromic match, F means forward (direst) match and R means reverse match

Table S2B. List of repeated sequences in the chloroplast genome of *Colobanthus affinis*

Repeat lenght (bp)	Strat site of repeat A	Repeat A location	Repeat A region	Strat site of repeat B	Repeat B location	Repeat B region	Repeat type
30	26881	*ndhA* (intron)	SSC	26892	*ndhA* (intron)	SSC	R
30	52356	*ycf2*	IR	52374	*ycf2*	IR	F
30	52354	*ycf2*	IR	52396	*ycf2*	IR	F
30	63572	IGS (*trnK-UUU-rps16*)	LSC	63576	IGS (*trnK-UUU-rps16*)	LSC	F
30	89089	IGS (*trnE-UUC-trnT-GGU*)	LSC	89121	IGS (*trnE-UUC-trnT-GGU*)	LSC	F
30	66850	*trnS-GCU*	LSC	93527	*trnS-UGA*	LSC	F
30	96696	*psaB*	LSC	98920	*psaA*	LSC	F
30	66850	*trnS-GCU*	LSC	103088	*trnS-GGA*	LSC	P
30	93527	*trnS-UGA*	LSC	103088	*trnS-GGA*	LSC	P
31	52377	*ycf2*	IR	52401	*ycf2*	IR	F
31	63572	IGS (*trnK-UUU-rps16*)	LSC	66696	IGS (*psbI-trnS-GCU*)	LSC	P
31	68460	*trnG-UCC*	LSC	94460	*trnG-GCC*	LSC	F
32	63572	IGS (*trnK-UUU-rps16*)	LSC	63574	IGS (*trnK-UUU-rps16*)	LSC	F
34	89077	IGS (*trnE-UUC-trnT-GGU*)	LSC	89109	IGS (*trnE-UUC-trnT-GGU*)	LSC	F
38	87263	IGS (*petN-psbM*)	LSC	87263	IGS (*petN-psbM*)	LSC	P
40	26641	*ndhA* (intron)	SSC	45918	IGS (*trnV-GAC-rps7*)	IR	P
41	19979	IGS (*rpl32-trnL-UAG*)	SSC	20034	IGS (*rpl32-trnL-UAG)*	SSC	F
41	33244	*ycf1*	SSC	33244	*ycf1*	SSC	R
61	19959	IGS (*rpl32-trnL-UAG*)	SSC	20014	IGS (*rpl32-trnL-UAG*)	SSC	F
103	100519	IGS (*psaA-ycf3*)	LSC	118296	IGS (*psaI-ycf4*)	LSC	P
134	100488	IGS (*psaA-ycf3*)	LSC	118296	IGS (*psaI-ycf4*)	LSC	P
169	100453	IGS (*psaA-ycf3*)	LSC	118296	IGS (*psaI-ycf4*)	LSC	P

IGS (*trnK-UUU-rps16*) means spacer between *trnK-UUU* and *rps16*, P means palindromic match, F means forward (direst) match and R means reverse match

Table S2C. List of repeated sequences in the chloroplast genome of *Colobanthus lycopodioides*

Repeat lenght (bp)	Strat site of repeat A	Repeat A location	Repeat A region	Strat site of repeat B	Repeat B location	Repeat B region	Repeat type
30	52273	*ycf2*	IR	52315	*ycf2*	IR	F
30	52275	*ycf2*	IR	52293	*ycf2*	IR	F
30	63516	IGS (*trnK-UUU-rps16*)	LSC	63518	IGS (*trnK-UUU-rps16*)	LSC	F
30	63516	IGS (*trnK-UUU-rps16*)	LSC	63520	IGS (*trnK-UUU-rps16*)	LSC	F
30	63615	IGS (*trnK-UUU-rps16*)	LSC	110958	IGS (*trnM-CAU-atpE*)	LSC	P
30	63615	IGS (*trnK-UUU-rps16*)	LSC	130085	*clpP* (intron1)	LSC	F
30	66856	*trnS-GCU*	LSC	93695	*trnS-UGA*	LSC	F
30	66856	*trnS-GCU*	LSC	103222	*trnS-GGA*	LSC	P
30	93695	*trnS-UGA*	LSC	103222	*trnS-GGA*	LSC	P
30	96869	*psaB*	LSC	99093	*psaA*	LSC	F
30	100720	IGS (*psaA-ycf3*)	LSC	118440	IGS (*psaI-ycf4*)	LSC	P
30	122218	IGS (*petA-psbJ*)	LSC	122219	IGS (*petA-psbJ*)	LSC	F
31	52296	*ycf2*	IR	52320	*ycf2*	IR	F
31	68472	*trnG-UCC*	LSC	94640	*trnG-GCC*	LSC	F
31	71794	IGS (*atpF-atpH*)	LSC	71834	IGS (*atpF-atpH*)	LSC	P
38	87379	IGS (*petN-psbM*)	LSC	87379	IGS (*petN-psbM*)	LSC	P
40	26595	*ndhA* (intron)	SSC	45837	IGS (*trnV-GAC-rps7*)	IR	P
41	33164	*ycf1*	SSC	33164	*ycf1*	SSC	R
42	63403	IGS (*trnK-UUU-rps16*)	LSC	63443	IGS (*trnK-UUU-rps16*)	LSC	F
134	100616	IGS (*psaA-ycf3*)	LSC	118440	IGS (*psaI-ycf4*)	LSC	P
169	100581	IGS (*psaA-ycf3*)	LSC	118440	IGS (*psaI-ycf4*)	LSC	P

IGS (*trnK-UUU-rps16*) means spacer between *trnK-UUU* and *rps16*, P means palindromic match, F means forward (direst) match and R means reverse match

Table S2D. List of repeated sequences in the chloroplast genome of *Colobanthus nivicola*

Repeat lenght (bp)	Strat site of repeat A	Repeat A location	Repeat A region	Strat site of repeat B	Repeat B location	Repeat B region	Repeat type
30	19876	IGS (*rpl32-trnL-UAG*)	SSC	19897	IGS (*rpl32-trnL-UAG*)	SSC	F
30	52316	*ycf2*	IR	52334	*ycf2*	IR	F
30	52314	*ycf2*	IR	52356	*ycf2*	IR	F
30	89058	IGS (*trnE-UUC-trnT-GGU*)	LSC	89090	IGS (*trnE-UUC-trnT-GGU*)	LSC	F
30	66802	*trnS-GCU*	LSC	93487	*trnS-UGA*	LSC	F
30	96649	*psaB*	LSC	98873	*psaA*	LSC	F
30	66802	*trnS-GCU*	LSC	103043	*trnS-GGA*	LSC	P
30	93487	*trnS-UGA*	LSC	103043	*trnS-GGA*	LSC	P
30	110793	IGS (*trnM-CAU-atpE*)	LSC	110793	IGS (*trnM-CAU-atpE*)	LSC	R
30	74728	IGS (*rps2-rpoC2*)	LSC	110794	IGS (*trnM-CAU-atpE*)	LSC	F
31	52337	*ycf2*	IR	52361	*ycf2*	IR	F
31	63536	IGS (*trnK-UUU-ndhA*)	LSC	63540	IGS (*trnK-UUU-ndhA*)	LSC	F
31	87862	IGS (*psbB-trnD-GUC*)	LSC	87862	IGS (*psbB-trnD-GUC*)	LSC	R
31	68413	*trnG-UCC*	LSC	94420	*trnG-GCC*	LSC	F
33	63536	IGS (*trnK-UUU-ndhA*)	LSC	63538	IGS (*trnK-UUU-ndhA*)	LSC	F
34	89046	IGS (*trnE-UUC-trnT-GGU*)	LSC	89078	IGS (*trnE-UUC-trnT-GGU*)	LSC	F
38	87230	IGS (*petN-psbM*)	LSC	87230	IGS (*petN-psbM*)	LSC	P
40	26617	*ndhA* (intron)	SSC	45878	IGS (*trnV-GAC-rps7*)	IR	P
41	33199	*ycf1*	SSC	33199	*ycf1*	SSC	R
97	100478	IGS (*psaA-ycf3*)	LSC	118250	IGS (*psaI-ycf4*)	LSC	P
134	100441	IGS (*psaA-ycf3*)	LSC	118250	IGS (*psaI-ycf4*)	LSC	P
169	100406	IGS (*psaA-ycf3*)	LSC	118250	IGS (*psaI-ycf4*)	LSC	P

IGS (*rpl32-trnL-UAG*) means spacer between *rpl32* and *trnL-UAG*, P means palindromic match, F means forward (direst) match and R means reverse match

Table S2E. List of repeated sequences in the chloroplast genome of *Colobanthus pulvinatus*

Repeat lenght (bp)	Strat site of repeat A	Repeat A location	Repeat A region	Strat site of repeat B	Repeat B location	Repeat B region	Repeat type
30	52284	*ycf2*	IR	52326	*ycf2*	IR	F
30	52286	*ycf2*	IR	52304	*ycf2*	IR	F
30	66692	*trnS-GCU*	LSC	93377	*trnS-UGA*	LSC	F
30	66692	*trnS-GCU*	LSC	102933	*trnS-GGA*	LSC	P
30	74474	IGS (*rps2-rpoC2*)	LSC	129737	*clpP* (intron1)	LSC	C
30	74605	IGS (*rps2-rpoC2*)	LSC	129736	*clpP* (intron1)	LSC	C
30	74618	IGS (*rps2-rpoC2*)	LSC	110690	IGS (*trnM-CAU-atpE*)	LSC	F
30	87750	IGS (*psbM-trnD-GUC*)	LSC	129743	*clpP* (intron1)	LSC	F
30	96539	*psaB*	LSC	98763	*psaA*	LSC	F
30	110686	IGS (*trnM-CAU-atpE*)	LSC	129736	*clpP* (intron1)	LSC	P
31	52307	*ycf2*	IR	52331	*ycf2*	IR	F
31	63506	IGS (*trnK-UUU-rps16*)	LSC	63512	IGS (*trnK-UUU-rps16*)	LSC	F
31	68303	*trnG-UCC*	LSC	94310	*trnG-GCC*	LSC	F
31	74611	IGS (rps2-rpoC2)	LSC	110683	IGS (*trnM-CAU-atpE*)	LSC	R
31	87752	IGS (*psbM-trnD-GUC*)	LSC	87752	IGS (*psbM-trnD-GUC*)	LSC	R
31	87752	IGS (*psbM-trnD-GUC*)	LSC	110684	IGS (*trnM-CAU-atpE*)	LSC	C
31	87752	IGS (*psbM-trnD-GUC*)	LSC	110684	IGS (*trnM-CAU-atpE*)	LSC	P
31	87752	IGS (*psbM-trnD-GUC*)	LSC	129737	*clpP* (intron1)	LSC	R
31	87752	IGS (*psbM-trnD-GUC*)	LSC	129737	*clpP* (intron1)	LSC	F
31	87753	IGS (*psbM-trnD-GUC*)	LSC	110685	IGS (*trnM-CAU-atpE*)	LSC	P
31	87753	IGS (*psbM-trnD-GUC*)	LSC	110686	IGS (*trnM-CAU-atpE*)	LSC	C
31	110675	IGS (*trnM-CAU-atpE*)	LSC	129737	*clpP* (intron1)	LSC	C
31	110683	IGS (*trnM-CAU-atpE*)	LSC	129735	*clpP* (intron1)	LSC	P
31	110686	IGS (*trnM-CAU-atpE*)	LSC	110689	IGS (*trnM-CAU-atpE*)	LSC	F
31	110688	IGS (*trnM-CAU-atpE*)	LSC	129739	*clpP* (intron1)	LSC	P
31	110691	IGS (*trnM-CAU-atpE*)	LSC	129745	*clpP* (intron1)	LSC	C
32	74615	IGS (*rps2-rpoC2*)	LSC	110683	IGS (*trnM-CAU-atpE*)	LSC	F

32	110680	IGS (*trnM-CAU-atpE*)	LSC	129736	*clpP* (intron1)	LSC	C
33	63506	IGS (*trnK-UUU-rps16*)	LSC	63510	IGS (*trnK-UUU-rps16*)	LSC	F
33	129741	*clpP* (intron1)	LSC	129741	*clpP* (intron1)	LSC	R
34	88936	IGS (*trnE-UCC-trnT-GGU*)	LSC	88968	IGS (*trnE-UCC-trnT-GGU*)	LSC	F
35	63506	IGS (*trnK-UUU-rps16*)	LSC	63508	IGS (*trnK-UUU-rps16*)	LSC	F
36	110683	IGS (*trnM-CAU-atpE*)	LSC	110683	IGS (*trnM-CAU-atpE*)	LSC	R
38	87120	IGS (*petN-psbM*)	LSC	87120	IGS (*petN-psbM*)	LSC	P
40	26587	*ndhA* (intron)	SSC	45848	*IGS (trnV-GAC-rps7)*	IR	P
41	33169	*ycf1*	SSC	33169	*ycf1*	SSC	R
97	100368	IGS (*psaA-ycf3*)	LSC	118146	IGS (*psaI-ycf4*)	LSC	P
134	100331	IGS (*psaA-ycf3*)	LSC	118146	IGS (*psaI-ycf4*)	LSC	P
169	100296	IGS (*psaA-ycf3*)	LSC	118146	IGS (*psaI-ycf4*)	LSC	P

IGS (*psbM-trnD-GUC*) means spacer between *psbM* and *trnD-GUC*, P means palindromic match, F means forward (direst) match, R means reverse match and C complementary match

Table S2F. List of repeated sequences in the chloroplast genome of *Colobanthus subulatus*

Repeat lenght (bp)	Strat site of repeat A	Repeat A location	Repeat A region	Strat site of repeat B	Repeat B location	Repeat B region	Repeat type
30	52284	*ycf2*	IR	52302	*ycf2*	IR	F
30	52282	*ycf2*	IR	52324	*ycf2*	IR	F
30	59167	IGS (*trnH-GUG-psbA*)	LSC	59199	IGS (*trnH-GUG-psbA*)	LSC	P
30	66912	*trnS-GCU*	LSC	93571	*trnS-UGA*	LSC	F
30	96733	*psaB*	LSC	98957	*psaA*	LSC	F
30	66912	*trnS-GCU*	LSC	103120	*trnS-GGA*	LSC	P
30	93571	*trnS-UGA*	LSC	103120	*trnS-GGA*	LSC	P
30	110857	IGS (*trnM-CAU-atpE*)	LSC	110858	IGS (*trnM-CAU-atpE*)	LSC	F
30	100630	IGS (*psaA-ycf3*)	LSC	118319	IGS (*psaI-ycf4*)	LSC	P
31	52305	*ycf2*	IR	52329	*ycf2*	IR	F
31	64323	*rps16* (intron)	LSC	64415	*rps16* (intron)	LSC	F
31	68528	*trnG-UCC*	LSC	94504	*trnG-GCC*	LSC	F
38	87341	IGS (*petN-psbM*)	LSC	87341	IGS (*petN-psbM*)	LSC	P
40	26604	*ndhA* (intron)	SSC	45846	IGS (*trnV-GAC-rps7*)	IR	P
41	33172	*ycf1*	SSC	33172	*ycf1*	SSC	R
42	108814	IGS (*ndhC-trnV-UAC*)	LSC	108856	IGS (*ndhC-trnV-UAC*)	LSC	F
47	64326	*rps16* (intron)	LSC	64395	*rps16* (intron)	LSC	F
70	64326	*rps16* (intron)	LSC	64372	*rps16* (intron)	LSC	F
93	64326	*rps16* (intron)	LSC	64349	*rps16* (intron)	LSC	F
134	100526	IGS (*psaA-ycf3*)	LSC	118319	IGS (*psaI-ycf4*)	LSC	P
169	100491	IGS (*psaA-ycf3*)	LSC	118319	IGS (*psaI-ycf4*)	LSC	P

IGS (*trnH-GUG-psbA*) means spacer between *trnH-GUG* and *psbA*, P means palindromic match, F means forward (direst) match and R means reverse match
