# Supplementary material for: Evolutionary dynamics of the chloroplast genome sequences of six Colobanthus species
Source: Sci Rep. 2020 Jul 13;10:11522. doi: 10.1038/s41598-020-68563-5 (PMC7359349; doi:10.1038/s41598-020-68563-5)
Supplement: Supplementary file 4 — Supplementary Table S3. [file 41598_2020_68563_MOESM4_ESM.pdf]

Evolutionary dynamics of the chloroplast genome sequences of six *Colobanthus* species

Piotr Androsiuk*[1], Jan Paweł Jastrzębski[1], Łukasz Paukszto[1], Karol Makowczenko[1], Adam Okorski[2], Agnieszka Pszczółkowska[2], Katarzyna Joanna Chwedorzewska[3], Ryszard Górecki[1], Irena Giełwanowska[1]

1. Department of Plant Physiology, Genetics and Biotechnology, Faculty of Biology and Biotechnology, University of Warmia and Mazury in Olsztyn, ul. M. Oczapowskiego 1A, 10-719 Olsztyn, Poland.
2 Department of Entomology, Phytopathology and Molecular Diagnostics, Faculty of Environmental Management and Agriculture, University of Warmia and Mazury in Olsztyn, ul. Prawocheńskiego 17, 10-720 Olsztyn, Poland.
3 Department of Agronomy, Warsaw University of Life Sciences-SGGW, ul. Nowoursynowska 166, 02-787 Warszawa, Poland.

Table S3A. Distribution of SSR in the *Colobanthus acicularis* cp genome.

Type	Repeat unit	Length (bp)	Start	End	Location
Mononucleotide	A	12	31 124	31 135	*ycf1*
		14	32 047	32 060	*ycf1*
		12	60 849	60 860	*trnK-UUU*
		17	63 539	63 555	IGS *(trnK-UUU-rps16)*
		12	65 850	65 861	IGS *(trnQ-UUG-psbK)*
		15	65 896	65 910	IGS *(trnQ-UUG-psbK)*
		13	66 485	66 497	IGS *(psbK-psbI)*
		14	71 130	71 143	*atpF* (intron)
		18	74 579	74 596	IGS *(rps2-rpoC2)*
		15	74 712	74 726	IGS *(rps2-rpoC2)*
		12	76 784	76 795	*rpoC2*
		23	87 819	87 841	IGS *(psbM-trnD-GUC)*
		17	89 208	89 224	IGS *(trnE-UUC-trnT-GGU)*
		13	90 483	90 495	IGS *(trnT-GGU-psbD)*
		16	93 106	93 121	IGS *(psbC-trnS-UGA)*
		13	106 246	106 258	IGS *(trnF-GAA-ndhJ)*
		14	114 876	114 889	IGS *(rbcL-accD)*
		13	123 654	123 666	IGS *(psbE-petL)*
		15	123 857	123 871	IGS *(psbE-petL)*
		12	127 886	127 897	IGS *(rpl20-rps12)*
		24	129 670	129 693	*clpP* (intron1)
		12	133 706	133 717	*petB* (intron)
Dinucleotide	AT	13	63 527	63 539	IGS *(trnK-UUU-rps16)*
		12	117 293	117 304	IGS *(accD-psaI)*
Trinucleotide	AAT	12	20 533	20 544	IGS *(trnL-UAG-ccsA)*
		13	103 784	103 796	IGS *(rps4-trnT-UGU)*
		12	137 244	137 255	IGS *(rpoA-rps11)*
Tetranucleotide	AAAT	12	18 715	18 726	IGS *(ndhF-rpl32)*
	AATT	12	27 329	27 340	*ndhA* (intron)
	ACCT	14	38 520	38 533	*rrn23*
	AATT	12	63 758	63 769	IGS *(trnK-UUU-rps16)*
	AATG	13	68 446	68 458	IGS *(trnG-UCC-trnR-UCU)*
	AAAC	12	68 883	68 894	*atpA*
	AAGG	13	71 794	71 806	IGS *(atpF-atpH)*
	AAAT	16	87 164	87 179	IGS *(petN-psbM)*
	AAAC	13	93 968	93 980	IGS *(psbZ-trnG-GCC)*
	AACT	13	100 123	100 135	IGS *(psaA-ycf3)*
	AATT	12	117 617	117 628	IGS *(accD-psaI)*
	AAAG	12	128 669	128 680	*clpP* (intron2)
	AAAT	12	135 112	135 123	*petD* (intron)
Pentanucleotide	AAATT	18	19 803	19 820	IGS *(rpl32-trnL-UAG)*
	AAATC	16	24 409	24 424	IGS *(ndhE-ndhG)*
	AATCT	15	123 764	123 778	IGS *(psbE-petL)*
Hexanucleotide	AATACT	25	94 365	94 389	IGS *(trnG-GCC-trnfM-CAU)*

IGS (*trnK-UUU-rps16*) means spacer between *trnK-UUU* and *rps16*

Table S3B. Distribution of SSR in the *Colobanthus affinis* cp genome.

Type	Repeat unit	Length (bp)	Start	End	Location
Mononucleotide	A	13	16 448	16 460	*ndhF*
		12	31 163	31 174	*ycf1*
		13	60 894	60 906	*trnK-UUU* (intron)
		16	63 591	63 606	IGS *(trnK-UUU-rps16)*
		20	65 946	65 965	IGS *(trnQ-UUG-psbK)*
		13	66 540	66 552	IGS *(psbK-psbI)*
		18	74 630	74 647	IGS *(rps2-rpoC2)*
		15	74 763	74 777	IGS *(rps2-rpoC2)*
		12	76 835	76 846	*rpoC2*
		18	89 308	89 325	IGS *(trnE-UUC-trnT-GGU)*
		12	90 745	90 756	IGS *(trnT-GGU-psbD)*
		13	93 367	93 379	IGS *(psbC-trnS-UGA)*
		14	106 515	106 528	IGS *(trnF-GAA-ndhJ)*
		14	115 142	115 155	IGS *(rbcL-accD)*
		14	124 100	124 113	IGS *(psbE-petL)*
		12	125 281	125 292	IGS *(trnP-UGG-psaJ)*
		12	128 147	128 158	IGS *(rpl20-clpP)*
		21	129 933	129 953	*clpP* (intron1)
		13	133 963	133 975	*petB* (intron)
Dinucleotide	AT	19	63 573	63 591	IGS *(trnK-UUU-rps16)*
		21	66 709	66 729	IGS *(psbI-trnS-GCU)*
		12	117 559	117 57	IGS *(accD-psaI)*
Trinucleotide	AAT	12	20 579	20 590	IGS *(trnL-UAG-ccsA)*
		13	104 064	104 076	IGS *(rps4-trnT-UGU)*
		12	137 508	137 519	IGS *(rpoA-rps11)*
Tetranucleotide	AAAT	12	18 722	18 733	IGS *(ndhF-rpl32)*
	AATT	12	27 367	27 378	*ndhA* (intron)
	ACCT	14	38 560	38 573	*rrn23*
	AATT	12	63 811	63 822	IGS *(trnK-UUU-rps16)*
	AAAC	12	68 942	68 953	*atpA*
	AAGG	13	71 854	71 866	IGS *(atpF-atpH)*
	AAAT	12	87 240	87 251	IGS *(petN-psbM)*
	AAAC	13	94 225	94 237	IGS *(psbZ-trnG-GCC)*
	AACT	13	100 407	100 419	IGS *(psaA-ycf3)*
	AATT	12	117 883	117 894	IGS *(accD-psaI)*
	AAAG	12	128 930	128 941	*clpP* (intron2)
	AAAT	12	135 376	135 387	*petD* (intron)
Pentanucleotide	AAATC	16	24 429	24 444	IGS *(ndhE-ndhG)*
	AATCT	15	124 007	124 021	IGS *(psbE-petL)*

IGS (*trnK-UUU-rps16*) means spacer between *trnK-UUU* and *rps16*

Table S3C. Distribution of SSR in the *Colobanthus lycopodioides* cp genome.

Type	Repeat unit	Length (bp)	Start	End	Location
Mononucleotide	A	15	18 781	18 795	IGS *(ndhF-rpl32)*
		12	31 083	31 094	*ycf1*
		13	60 790	60 802	*trnK-UUU* (intron)
		14	63 535	63 548	IGS *(trnK-UUU-rps16)*
		14	63 627	63 640	IGS *(trnK-UUU-rps16)*
		14	65 932	65 945	IGS *(trnQ-UUG-psbK)*
		14	65 980	65 993	IGS *(trnQ-UUG-psbK)*
		12	66 570	66 581	IGS *(psbK-psbI)*
		13	68 787	68 799	IGS *(trnR-UCU-atpA)*
		16	71 214	71 229	*atpF* (intron)
		12	71 284	71 295	*atpF* (intron)
		14	74 747	74 760	IGS *(rps2-rpoC2)*
		17	74 879	74 895	IGS *(rps2-rpoC2)*
		12	76 944	76 955	*rpoC2*
		13	89 485	89 497	IGS *(trnE-UUC-trnT-GGU)*
		12	90 912	90 923	IGS *(trnT-GGU-psbD)*
		14	93 534	93 547	IGS *(psbC-trnS-UGA)*
		12	93 985	93 996	IGS *(trnS-UGA-psbZ)*
		12	102 560	102 571	IGS *(ycf3-trnS-GGA)*
		12	109 212	109 223	IGS *(ndhC-trnV-UAC)*
		18	122 231	122 248	IGS *(petA-psbJ)*
		16	124 283	124 298	IGS *(psbE-petL)*
		12	128 306	128 317	IGS *(rpl20-clpP)*
		14	129 942	129 955	*clpP* (intron1)
		22	130 097	130 118	*clpP* (intron1)
		12	134 121	134 132	*petB* (intron)
Dinucleotide	AT	19	63 517	63 535	IGS *(trnK-UUU-rps16)*
		12	117 703	117 714	IGS *(accD-psaI)*
Trinucleotide	AAT	12	20 526	20 537	IGS *(trnL-UAG-ccsA)*
		13	104 198	104 210	IGS *(rps4-trnT-UGU)*
		12	137 659	137 670	IGS *(rpoA-rps11)*
Tetranucleotide	AAAT	12	18 721	18 732	IGS *(ndhF-rpl32)*
	AATT	12	27 287	27 298	*ndhA* (intron)
	ACCT	14	38 479	38 492	*rrn23*
	AATT	12	63 849	63 860	*rps16*
	AATG	13	68 521	68 533	IGS *(trnG-UCC-trnR-UCU)*
	AAAC	12	68 960	68 971	*atpA*
	AAGG	13	71 949	71 961	IGS *(atpF-atpH)*
	AAAT	12	87 356	87 367	IGS *(petN-psbM)*
	AAAC	13	94 404	94 416	IGS *(psbZ-trnG-GCC)*
	AACT	13	100 535	100 547	IGS *(psaA-ycf3)*
	AAAT	15	111 001	111 015	IGS *(trnM-CAU-atpE)*
	AATT	12	118 027	118 038	IGS *(accD-psaI)*
	AAAG	12	129 089	129 100	*clpP* (intron2)
	AAAT	12	135 527	135 538	*petD* (intron)
Pentanucleotide	AATTC	15	24 370	24 384	IGS *(ndhE-ndhG)*

	AATCT	15	124 190	124 204	IGS *(psbE-petL)*

IGS (*trnK-UUU-rps16*) means spacer between *trnK-UUU* and *rps16*

Table S3D. Distribution of SSR in the *Colobanthus nivicola* cp genome.

Type	Repeat unit	Length (bp)	Start	End	Location
Mononucleotide	A	13	16 453	16 465	*ndhF*
		12	18 787	18 798	IGS *(ndhF-rpl32)*
		12	31 118	31 129	*ycf1*
		12	32 338	32 349	*ycf1*
		15	60 855	60 869	*trnK-UUU*
		19	63 553	63 571	IGS *(trnK-UUU-rps16)*
		15	63 639	63 653	IGS *(trnK-UUU-rps16)*
		16	65 913	65 928	IGS *(trnQ-UUG-psbK)*
		12	66 503	66 514	IGS *(psbK-psbI)*
		12	67 187	67 198	IGS *(trnS-GCU-trnG-UCC)*
		21	74 593	74 613	IGS *(rps2-rpoC2)*
		17	74 729	74 745	IGS *(rps2-rpoC2)*
		12	76 803	76 814	*rpoC2*
		15	87 871	87 885	IGS *(psbM-trnD-GUC)*
		13	89 277	89 289	IGS *(trnE-UUC-trnT-GGU)*
		13	90 703	90 715	IGS *(trnT-GGU-psbD)*
		14	93 326	93 339	IGS *(psbC-trnS-UGA)*
		14	106 471	106 484	IGS *(trnF-GAA-ndhJ)*
		15	110 797	110 811	IGS *(trnM-CAU-atpE)*
		13	115 097	115 109	IGS *(rbcL-accD)*
		14	124 058	124 071	IGS *(psbE-petL)*
		13	128 096	128 108	IGS *(rpl20-rps12)*
		19	129 882	129 900	*clpP* (intron1)
		14	133 857	133 870	*petB*
Dinucleotide	AT	17	63 537	63 553	IGS *(trnK-UUU-rps16)*
		12	117 513	117 524	IGS *(accD-psaI)*
Trinucleotide	AAT	12	20 555	20 566	IGS *(trnL-UAG-ccsA)*
		13	104 019	104 031	IGS *(rps4-trnT-UGU)*
		12	137 403	137 414	IGS *(rpoA-rps11)*
Tetranucleotide	AAAT	12	18 727	18 738	IGS *(ndhF-rpl32)*
	AATT	12	27 322	27 333	*ndhA*
	ACCT	14	38 514	38 527	*rrn23*
	AATT	12	63 855	63 866	IGS *(trnK-UUU-rps16)*
	AAAC	12	68 895	68 906	*atpA*
	AAGG	13	71 808	71 820	IGS *(atpF-atpH)*
	AAAT	12	87 207	87 218	IGS *(petN-psbM)*
	AAAC	13	94 185	94 197	IGS *(psbZ-trnG-GCC)*
	AACT	13	100 360	100 372	IGS *(psaA-ycf3)*
	AATT	12	117 837	117 848	IGS *(accD-psaI)*
	AAAG	12	128 880	128 891	*clpP* (intron2)
	AAAT	12	135 271	135 282	*petD*
Pentanucleotide	AAATT	18	19 816	19 833	IGS *(rpl32-trnL-UAG)*
	AAATC	16	24 405	24 420	IGS *(ndhE-ndhG)*
	AATCT	15	123 965	123 979	IGS *(psbE-petL)*
Hexanucleotide	AAGAAT	19	41 278	41 296	IGS *(trnA-UGC-trnI-GAU)*

IGS (*trnK-UUU-rps16*) means spacer between *trnK-UUU* and *rps16*

Table S3E. Distribution of SSR in the *Colobanthus pulvinatus* cp genome.

Type	Repeat unit	Length (bp)	Start	End	Location
Mononucleotide	A	13	16 444	16 456	*ndhF*
		12	18 778	18 789	IGS *(ndhF-rpl32)*
		12	31 088	31 099	*ycf1*
		12	32 308	32 319	*ycf1*
		15	60 825	60 839	*trnK-UUU* (intron)
		15	63 529	63 543	IGS *(trnK-UUU-rps16)*
		16	65 803	65 818	IGS *(trnQ-UUG-psbK)*
		12	66 393	66 404	IGS *(psbK-psbI)*
		12	67 077	67 088	IGS *(trnS-GCU-trnG-UCC)*
		21	74 483	74 503	IGS *(rps2-rpoC2)*
		17	74 619	74 635	IGS *(rps2-rpoC2)*
		12	76 693	76 704	*rpoC2*
		15	87 761	87 775	IGS *(psbM-trnD-GUC)*
		13	89 167	89 179	IGS *(trnE-UUC-trnT-GGU)*
		13	90 593	90 605	IGS *(trnT-GGU-psbD)*
		14	93 216	93 229	IGS *(psbC-trnS-UGA)*
		14	106 361	106 374	IGS *(trnF-GAA-ndhJ)*
		21	110 687	110 707	IGS *(trnM-CAU-atpE)*
		13	114 993	115 005	IGS *(rbcL-accD)*
		14	123 954	123 967	IGS *(psbE-petL)*
		13	127 963	127 975	IGS *(rpl20-clpP)*
		23	129 746	129 768	*clpP* (intron1)
		14	133 780	133 793	*petB* (intron)
Dinucleotide	AT	23	63 507	63 529	IGS *(trnK-UUU-rps16)*
		12	117 409	117 420	IGS *(accD-psaI)*
Trinucleotide	AAT	12	20 525	20 536	IGS *(trnL-UAG-ccsA)*
		13	103 909	103 921	IGS *(rps4-trnT-UGU)*
		12	137 326	137 337	*rpoA*
Tetranucleotide	AAAT	12	18 718	18 729	IGS *(ndhF-rpl32)*
	AATT	12	27 292	27 303	*ndhA* (intron)
	ACCT	14	38 484	38 497	*rrn23*
	AATT	12	63 745	63 756	IGS *(trnK-UUU-rps16)*
	AAAC	12	68 785	68 796	*atpA*
	AAGG	13	71 698	71 710	IGS *(atpF-atpH)*
	AAAT	12	87 097	87 108	IGS *(petN-psbM)*
	AAAC	13	94 075	94 087	IGS *(psbZ-trnG-GCC)*
	AACT	13	100 250	100 262	IGS *(psaA-ycf3)*
	AATT	12	117 733	117 744	IGS *(accD-psaI)*
	AAAG	12	128 747	128 758	*clpP* (intron2)
	AAAT	12	135 194	135 205	*petD* (intron)
Pentanucleotide	AAATT	18	19 807	19 824	IGS *(rpl32-trnL-UAG)*
	AAATC	16	24 375	24 390	IGS *(ndhE-ndhG)*
	AATCT	15	123 861	123 875	IGS *(psbE-petL)*
Hexanucleotide	AAGAAT	19	41 248	41 266	IGS *(trnA-UGC-trnI-GAU)*

IGS (*trnK-UUU-rps16*) means spacer between *trnK-UUU* and *rps16*

Table S3F. Distribution of SSR in the *Colobanthus subulatus* cp genome.

Type	Repeat unit	Length (bp)	Start	End	Location
Mononucleotide	A	13	16 449	16 461	*ndhF*
		12	31 091	31 102	*ycf1*
		12	63 503	63 514	IGS *(trnK-UUU-rps16)*
		12	63 582	63 593	IGS *(trnK-UUU-rps16)*
		15	66 030	66 044	IGS *(trnQ-UUG-psbK)*
		14	66 620	66 633	IGS *(psbK-psbI)*
		15	74 836	74 850	IGS *(atpI-rps2)*
		12	76 908	76 919	*rpoC2*
		14	87 983	87 996	IGS *(psbM-trnD-GUC)*
		16	88 162	88 177	IGS *(psbM-trnD-GUC)*
		16	89 360	89 375	IGS *(trnE-UUC-trnT-GGU)*
		12	104 721	104 732	IGS *(trnT-UGU-trnL-UAA)*
		15	106 496	106 510	IGS *(trnF-GAA-ndhJ)*
		18	110 858	110 875	IGS *(trnM-CAU-atpE)*
		13	115 156	115 168	IGS *(rbcL-accD)*
		20	124 134	124 153	IGS *(psbE-petL)*
		17	124 542	124 558	IGS *(petL-petG)*
		15	129 919	129 933	*clpP* (intron1)
		13	133 944	133 956	*petB* (intron)
Dinucleotide	AT	13	63 491	63 503	IGS *(trnK-UUU-rps16)*
	AT	15	66 790	66 804	IGS *(psbI-trnS-GCU)*
	AT	12	117 582	117 593	IGS *(accD-psaI)*
Trinucleotide	AAT	12	20 530	20 541	IGS *(trnL-UAG-ccsA)*
	AAT	13	104 096	104 108	IGS *(rps4-trnT-UGU)*
	AAT	12	137 483	137 494	IGS *(rpoA-rps11)*
Tetranucleotide	AAAT	12	18 723	18 734	IGS *(ndhF-rpl32)*
	AATT	12	27 295	27 306	*ndhA* (intron)
	ACCT	14	38 488	38 501	*rrn23*
	AATT	12	63 801	63 812	*rps16*
	AATG	13	68 577	68 589	IGS *(trnG-UCC-trnR-UCU)*
	AAAC	12	69 012	69 023	*atpA*
	AAGG	13	71 925	71 937	IGS *(atpF-atpH)*
	AAAC	13	94 269	94 281	IGS *(psbZ-trnG-GCC)*
	AACT	13	100 444	100 456	IGS *(psaA-ycf3)*
	AATT	12	117 906	117 917	IGS *(accD-psaI)*
	AAAG	12	128 917	128 928	*clpP* (intron2)
	AAAT	12	135 351	135 362	*petD* (intron)
Pentanucleotide	AATCC	18	23 776	23 793	IGS *(psaC-ndhE)*
	AAATT	17	19 810	19 826	IGS *(rpl32-trnL-UAG)*
	AAATC	16	24 385	24 400	IGS *(ndhE-ndhG)*
	AATCT	15	124 041	124 055	IGS *(psbE-petL)*

IGS (*trnK-UUU-rps16*) means spacer between *trnK-UUU* and *rps16*
