# Supplementary material for: Evolutionary dynamics of the chloroplast genome sequences of six Colobanthus species
Source: Sci Rep. 2020 Jul 13;10:11522. doi: 10.1038/s41598-020-68563-5 (PMC7359349; doi:10.1038/s41598-020-68563-5)
Supplement: Supplementary file 7 — Supplementary Table S6. [file 41598_2020_68563_MOESM7_ESM.pdf]

Evolutionary dynamics of the chloroplast genome sequences of six *Colobanthus* species

Piotr Androsiuk*[1], Jan Paweł Jastrzębski[1], Łukasz Paukszto[1], Karol Makowczenko[1], Adam Okorski[2], Agnieszka Pszczółkowska[2], Katarzyna Joanna Chwedorzewska[3], Ryszard Górecki[1], Irena Giełwanowska[1]

1. Department of Plant Physiology, Genetics and Biotechnology, Faculty of Biology and Biotechnology, University of Warmia and Mazury in Olsztyn, ul. M. Oczapowskiego 1A, 10-719 Olsztyn, Poland.

2 Department of Entomology, Phytopathology and Molecular Diagnostics, Faculty of Environmental Management and Agriculture, University of Warmia and Mazury in Olsztyn, ul. Prawocheńskiego 17, 10-720 Olsztyn, Poland.

3 Department of Agronomy, Warsaw University of Life Sciences-SGGW, ul. Nowoursynowska 166, 02-787 Warszawa, Poland.

Table S6. RNA editing sites predicted in the CP genomes of eight *Colobanthus* species using the PREP program.

Gene	Nucleotide position	Amino acid position	Codon change	Amino acid change	Score
accD	74	25	ACG => ATG	T => M	1.00
	229	77	CCT => TCT	P => S	1.00
atpA	914	305	TCA => TTA	S => L	1.00
	1270	424	CCC => TCC	P => S	1.00
atpB	419	140	ACA => ATA	T => I	0.86
atpF	–	–	–	–	–
atpI	–	–	–	–	–
ccsA	–	–	–	–	–
clpP	559	187	CAT => TAT	H => Y	1.00
matK	637	213	CAT => TAT	H => Y	1.00
ndhA	566	189	TCA => TTA	S => L	1.00
	1073	358	TCC => TTC	S => F	1.00
ndhB	149	50	TCA => TTA	S => L	1.00
	467[$]	156[$]	CCA => CTA	P => L	1.00
	542	181	ACG => ATG	T => M	1.00
	586	196	CAT => TAT	H => Y	1.00
	611	204	TCA => TTA	S => L	0.80
	737	246	CCA => CTA	P => L	1.00
	746	249	TCT => TTT	S => F	1.00
	830	277	TCA => TTA	S => L	1.00
	836	279	TCA => TTA	S => L	1.00
	1481	494	CCA => CTA	P => L	1.00
ndhD	29	10	ACG => ATG	T => M	1.00
	53	18	ACA => ATA	T => I	1.00
	94	32	CTT => TTT	L => F	1.00
	410	137	TCA => TTA	S => L	1.00
	637	213	CCG => TCG	P => S	1.00
	1325	442	TCA => TTA	S => L	0.80
	1337	446	TCA => TTA	S => L	0.80
	1366	456	CTT => TTT	L => F	0.80
	1424	475	ACT => ATT	T => I	1.00
	1487	496	GCT => GTT	A => V	0.80
ndhF	290	97	TCA => TTA	S => L	1.00
	586	196	CTT => TTT	L => F	0.80
	1769[£]	590[£]	CCT => CTT	P => L	0.80
ndhG	166	56	CAT => TAT	H => Y	0.80

	250	84	CCA => TCA	P => S	1.00
	314	105	ACA => ATA	T => I	0.80
petB	418	140	CGG => TGG	R => W	1.00
petD	–	–	–	–	–
petG	–	–	–	–	–
petL	–	–	–	–	–
psaB	–	–	–	–	–
psaI	28	10	CTT => TTT	L => F	0.86
psbB	–	–	–	–	–
psbE	–	–	–	–	–
psbF	–	–	–	–	–
psbL	17	6	ACG => ATG	T => M	1.00
rpl2	–	–	–	–	–
rpl20	–	–	–	–	–
rpoA	–	–	–	–	–
rpoB	169	57	CAT => TAT	H => Y	1.00
	473	158	TCA => TTA	S => L	0.86
	551	184	TCA => TTA	S => L	1.00
	566	189	TCG => TTG	S => L	1.00
	2033/2036*	678/679*	GCA => GTA	A => V	1.00
	2177/2180*	726/727*	GCG => GTG	A => V	1.00
rpoC1	41	14	TCA => TTA	S => L	1.00
rpoC2	1493	498	ACG => ATG	T => M	0.86
	2278	760	CGG => TGG	R => W	1.00
rps14	80	27	TCA => TTA	S => L	1.00
rps16	–	–	–	–	–
rps2	248	83	TCA => TTA	S => L	1.00
rps8	–	–	–	–	–
ycf3	–	–	–	–	–

$ - RNA editing site not observed for *C. lycopodioides*;
£ - RNA editing site not observed for *C. acicularis*;
*- RNA editing sites with shifted position revealed for *C. quitensis*
